# Structure and Cytotoxicity of Novel Lignans and Lignan Glycosides from the Aerial Parts of *Larrea tridentata*

**DOI:** 10.3390/molecules26206186

**Published:** 2021-10-14

**Authors:** Akihito Yokosuka, Tomoki Iguchi, Maki Jitsuno, Yoshihiro Mimaki

**Affiliations:** School of Pharmacy, Tokyo University of Pharmacy and Life Sciences, 1432-1, Horinouchi, Hachioji, Tokyo 192-0392, Japan; yokosuka@toyaku.ac.jp (A.Y.); sasatatesatoru@gmail.com (M.J.); mimakiy@toyaku.ac.jp (Y.M.)

**Keywords:** *Larrea tridentata*, Zygophyllaceae, aerial part, lignan, lignan glycoside, cytotoxicity, HL-60 cell, apoptosis, cell cycle

## Abstract

Previously, the authors conducted phytochemical investigations of the aerial parts of *Larrea tridentata* and reported triterpene glycosides and lignan derivatives. In continuation of the preceding studies, 17 lignans and lignan glycosides (**1**–**17**) were isolated, including seven new compounds (**1**–**7**). Herein, the structure of the new compounds was determined based on spectroscopic analysis and enzymatic hydrolysis. The cytotoxicity of **1**–**17** against HL-60 human promyelocytic leukemia cells was examined. Compounds **4**–**11** and **14**–**16** were cytotoxic to HL-60 cells, with IC_50_ values in the range of 2.7–17 μM. Compound **6**, which was the most cytotoxic among the unprecedented compounds, was shown to induce apoptotic cell death in HL-60 cells.

## 1. Introduction

*Larrea tridentata* is an evergreen small shrub belonging to the Zygophyllaceae family. It grows in the desert areas of the southwestern United States and Mexico, and is commonly called ‘Creosote bush’. It has been used as a medicinal plant for the treatment of a variety of illnesses—including infertility, rheumatism, arthritis, diabetes, gallbladder and kidney stones, pain, and inflammation [1]. The phytochemical constituents of *L. tridentata* have been extensively investigated with the aim of discovering new biologically active secondary metabolites. In this quest, bioactive compounds with antibacterial [2,3], antimicrobial [3], cytotoxic [2,4], anti-inflammatory [5], anti-tuberculosis [6], anti-fungal [7], and anti-protozoal [8] potential have been isolated and identified. Previously, we reported 25 triterpene glycosides and three lignan derivatives, including larrealignans A and B, from the aerial parts of *L. tridentata*, and their cytotoxicity against HL-60 human promyelocytic leukemia cells [9,10]. Some plant lignans are promising seed compounds for new anticancer agents, and etoposide, a clinically applied anticancer medicine, is a chemically modified plant lignan. Acute promyelocytic leukemia is a cancer of the white blood cells, and there will be approximately 14,287 new cases and 8809 deaths in 2018 in Japan [11,12]. It is relatively sensitive to chemotherapy agents, often successfully leading to remission. However, when leukemia recurs, the present anticancer agents are not satisfactorily effective against leukemia. Thus, the development of new antileukemia agents is still expected to overcome leukemia. Our phytochemical investigation of this plant with the focus on lignan constituents resulted in the isolation of 17 lignan derivatives (**1**–**17**), seven (**1**–**7**) of which were previously undescribed. This study deals with the structural characterization of novel lignan derivatives based on spectroscopic analysis and hydrolysis. The cytotoxicity of the isolated compounds against HL-60 cells is evaluated.

## 2. Results and Discussion

### 2.1. Structure Elucidation of **1–17**

**Figure 1:**
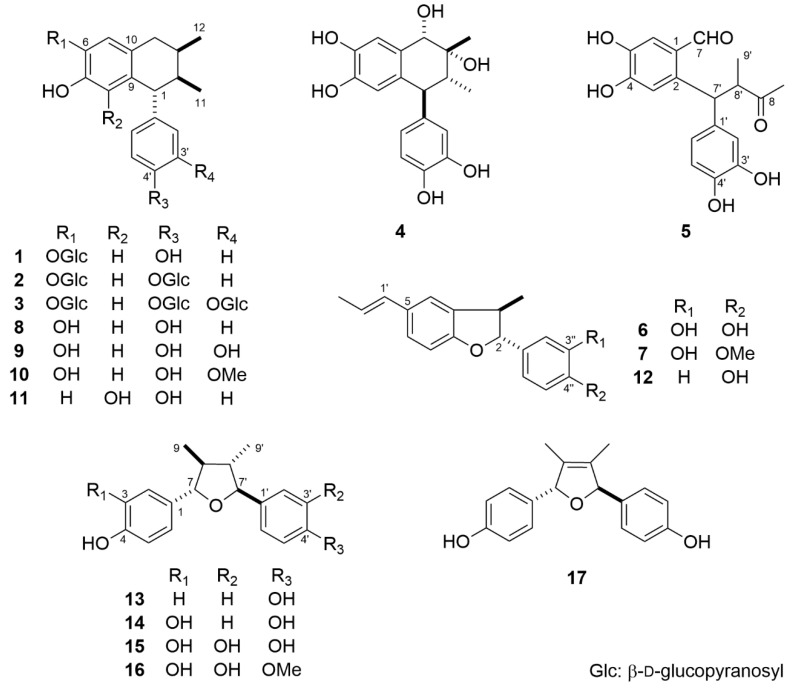
Structures of **1**–**17**.

The MeOH extract (940 g) of the aerial parts of *L. tridentata* (dry weight, 3.0 kg) was separated by silica gel and octadecylsilanized (ODS) silica gel column chromatography (CC) to collect 17 compounds (**1**–**17**) (Figure 1). Compounds **8**–**17** were identified as 3′-demethoxy-6-demethylisoguaiacin (**8**) [13], 6,3′-didemethylisoguaiacin (**9**) [14], 6-demethylisoguaiacin (**10**) [15], 6,3′-didemethoxy-8-hydroxyisoguaiacin (**11**) [16], (−)-conocarpan (**12**) [17], (−)-8′-*epi*-larreatricin (**13**) [18], (7*S*,7′*S*,8*S*,8′*S*)-3,4,4′-trihydroxy-7,7′-epoxylignan (**14**) [14], (7*S*,7′*S*,8*S*,8′*S*)-3,3′,4,4′-tetrahydroxy-7,7′-epoxylignan (**15**) [14], (7*S*,7′*S*,8*S*,8′*S*)-3,3′,4-trihydroxy-4′-methoxy-7,7′-epoxylignan (**16**) [19], and (7*R*,7′*R*)-4,4′-dihydroxy-8,8′-didehydro-7,7′-epoxylignan (**17**) [14].

Compound **1** was obtained as an amorphous powder. The molecular formula was determined as C_24_H_30_O_8_ based on high-resolution electrospray ionization time-of-flight mass spectroscopy (HR-ESI-TOF-MS; *m/z*: 469.1843 [M + Na]^+^, calculated for C_24_H_30_NaO_8_: 469.1838) and ^13^C-NMR. The ultraviolet (UV) spectrum of **1** exhibited an absorption maximum indicative of aromatic rings (281 nm). The ^1^H- and ^13^C-NMR spectra of **1** indicated the presence of a 1,2,4,5-tetrasubstituted aromatic ring [δ_H_ 6.97 (1H, s, H-5), 6.31 (1H, s, H-8); δ_C_ 116.9, 143.3, 144.2, 116.7, 133.2, 127.1 (C-5–C-10)], a 1,4-disubstituted aromatic ring [δ_H_ 6.85 (2H, d, *J* = 8.5 Hz, H-2′, H-6′), 6.71 (2H, d, *J* = 8.5 Hz, H-3′, H-5′); δ_C_ 137.4, 129.1, 113.9, 154.5, 113.9, 129.1 (C-1′–C-6′)], two methyl groups [δ_H_ 0.93 (3H, d, *J* = 7.0 Hz, Me-12), 0.91 (3H, d, *J* = 6.8 Hz, Me-11); δ_C_ 14.3 (C-11), 14.1 (C-12)], a methylene group [δ_H_ 2.90 (1H, dd, *J* = 16.0, 5.5 Hz, H-4a) and 2.49 (1H, dd, *J* = 16.0, 7.0 Hz, H-4b); δ_C_ 34.2 (C-4)], and three methine groups [δ_H_ 3.60 (1H, d, *J* = 6.6 Hz, H-1), 2.04 (1H, m, H-3), 1.94 (1H, ddd, *J* = 6.8, 6.6, 2.5 Hz, H-2); δ_C_ 49.4 (C-1), 40.2 (C-2), 28.7 (C-3)]. These spectral features are analogous to those of **8**. The glycosidic nature of **1** was corroborated by the strong absorption band of the hydroxy groups (3365 cm^−1^) in the infrared (IR) spectrum, and the signals of an anomeric proton and carbon [δ_H_ 4.78 (1H, d, *J* = 8.0 Hz, H-1″); δ_C_ 102.7 (C-1″)] in the ^1^H- and ^13^C-NMR spectra. Enzymatic hydrolysis of **1** with β-D-glucosidase in AcOH/AcONa buffer (pH 5.0) yielded **8** and D-glucose. D-Glucose was identified by HPLC analysis using a combination of refractive index and optical rotation detectors. In the heteronuclear multiple bond correlation (HMBC) spectrum of **1**, a long-range correlation was observed between H-1″ of β-D-glucopyranosyl (Glc) and C-6 of the aglycone moiety (δ_C_ 143.3). The β-anomeric configuration was ascertained based on the large coupling constant of the anomeric proton (*J* = 8.0 Hz). Therefore, **1** was characterized as 3′-demethoxy-6-demethylisoguaiacin 6-*O*-β-D-glucopyranoside.

The ^1^H- and ^13^C-NMR spectra of **2** (C_30_H_40_O_13_) were closely related to those of **1**; however, signals of an additional anomeric proton and carbon were observed. Furthermore, the molecular formula of **2** had an additional C_6_H_10_O_5_ compared to that of **1**. Thus, **2** was suggested to have one more hexopyranosyl group than **1**. Compound **2** was enzymatically hydrolyzed under the same conditions as those used for **1** to afford **8** and D-glucose. Analysis of the ^1^H-^1^H correlation spectroscopy (COSY), heteronuclear single quantum coherence (HSQC), 1-dimensional selective total correlation spectroscopy (1D-TOCSY), and HSQC-TOCSY data for the sugar moieties indicated the presence of two terminal β-D-glucopyranosyl units [Glc (I): δ_H_ 4.78 (1H, d, *J* = 7.9 Hz, H-1″ of Glc (I)); δ_C_ 102.7, 73.1, 76.1, 69.5, 76.2, 60.6 (C-1″–C-6″ of Glc (I))] and [Glc (II): δ_H_ 4.92 (1H, d, *J* = 7.9 Hz, H-1′″ of Glc (II)); δ_C_ 100.5, 73.1, 75.8, 69.5, 76.4, 60.6 (C-1′″–C-6′″ of Glc (II))]. The HMBC spectrum of **2** exhibited long-range correlations from H-1″ of Glc (I) to C-6 of the aglycone moiety (δ_C_ 143.4), and from H-1′″ of Glc (II) to C-4′ of the aglycone moiety (δ_C_ 155.4). Accordingly, **2** was identified as 3′-demethoxy-6-demethylisoguaiacin 6,4′-di-*O*-β-D-glucopyranoside.

Compound **3** (C_36_H_50_O_19_) was obtained as an amorphous powder. The ^1^H- and ^13^C-NMR spectra of **3** were similar to those of **2**, except for the signals assignable to the aromatic ring attached to C-1 of the aliphatic moiety. Signals arising from a 1,2,4-trisubstituted aromatic ring [δ_H_ 7.54 (1H, d, *J* = 8.3 Hz, H-5′), 7.51 (1H, br s, H-2′), 6.68 (1H, d, *J* = 8.3 Hz, H-6′); δ_C_ 143.0, 120.8, 148.3, 147.2, 118.9, 124.1 (C-1′–C-6′)] were observed in the ^1^H- and ^13^C-NMR spectra of **3**. Enzymatic hydrolysis of **3** with β-D-glucosidase yielded **9** and D-glucose. ^1^H-^1^H COSY, HSQC, 1D-TOCSY, and HSQC-TOCSY analyses of the sugar moieties revealed the following three terminal β-D-glucopyranosyl units: Glc (I) [δ_H_ 5.53 (1H, d, *J* = 8.0 Hz, H-1″ of Glc (I)); δ_C_ 105.1, 75.2, 78.3, 71.2, 79.0, 62.3 (C-1″–C-6″ of Glc (I))], Glc (II) [δ_H_ 5.62 (1H, d, *J* = 7.5 Hz, H-1′″ of Glc (II)); δ_C_ 103.8, 75.0, 78.3, 71.2, 78.5, 62.2 (C-1′″–C-6′″ of Glc (II))], and Glc (III) [δ_H_ 5.56 (1H, d, *J* = 7.4 Hz, H-1″″ of Glc (III)); δ_C_ 104.1, 75.1, 78.1, 71.2, 78.8, 62.3 (C-1″″–C-6″″ of Glc (III))]. The HMBC spectrum of **3** showed correlations between H-1″ of Glc (I) and C-6 of the aglycone moiety (δ_C_ 145.4), H-1′″ of Glc (II) and C-3′ of the aglycone moiety (δ_C_ 148.3), and between H-1″″ of Glc (III) and C-4′ of the aglycone moiety (δ_C_ 147.2). Thus, **3** was determined to be 6,3′-didemethylisoguaiacin 6,3′,4′-tri-*O*-β-D-glucopyranoside.

**Figure 2:**
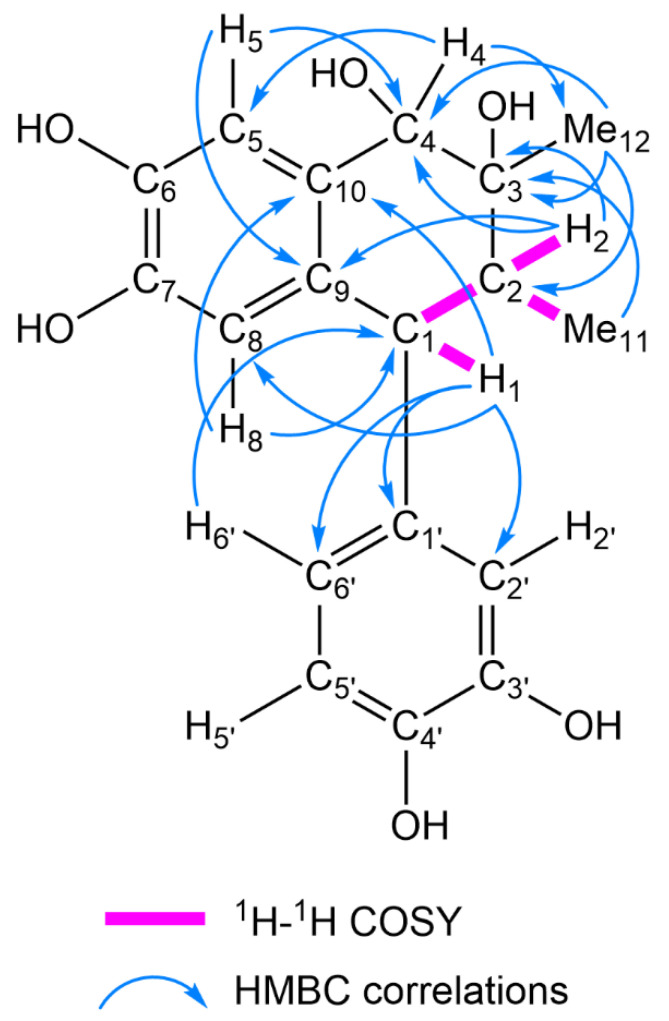
^1^H-^1^H COSY and key HMBC correlations of **4**.

**Figure 3:**
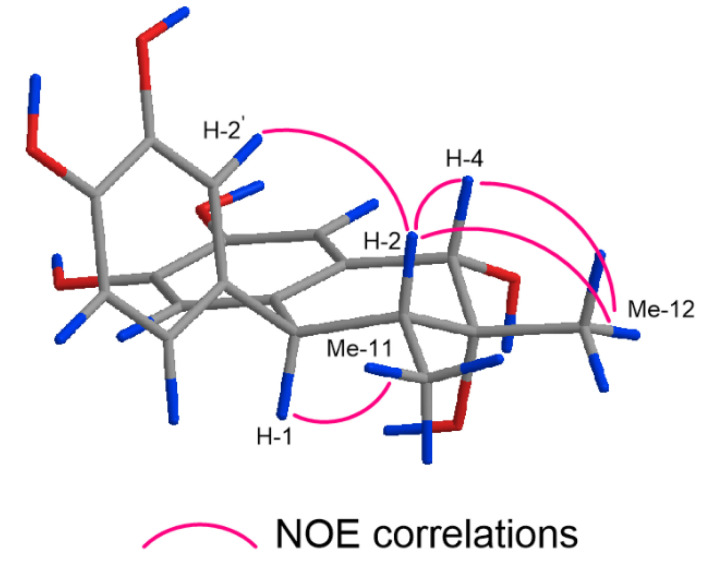
Key NOE correlations of **4**.

Compound **4** (C_18_H_20_O_6_) was obtained as an amorphous powder. The ^1^H- and ^13^C-NMR spectra of **4** were suggestive of a 1-aryl tetralin-type lignan closely related to **9**, and **4** and **9** shared the same 1,2,4-trisubstituted aromatic ring and 1,2,4,5-tetrasubstituted aromatic ring. Compound **4** differed from **9** in the aliphatic ring moiety; that of **4** was composed of two methyl groups [δ_H_ 1.41 (3H, s, Me-12), 0.92 (3H, d, *J* = 6.7 Hz, Me-11); 21.9 (C-12), 11.0 (C-11)], two methine groups [δ_H_ 3.56 (1H, d, *J* = 11.0 Hz, H-1), 1.88 (1H, dd, *J* = 11.0, 6.7 Hz, H-2); δ_C_ 48.3 (C-1), 43.6 (C-2)], an oxygenated methine group [δ_H_ 4.48 (1H, s, H-4); δ_C_ 73.5 (C-4)], and an oxygenated quaternary carbon [δ_C_ 72.3 (C-3)]. The ^1^H-^1^H COSY spectrum of **4** indicated spin-coupling correlations of the H-2 methine proton with the H-1 methine proton and Me-11 methyl protons. The HMBC spectrum of **4** indicated long-range correlations from H-2 to C-3 and C-4, Me-12 to C-2, C-3, and C-4, and from H-4 to C-12. These correlations indicated the introduction of hydroxy groups at C-3 and C-4 (Figure 2). Thus, the planar structure of **4** was determined to be 1,2,4-trihydro-1-(3,4-dihydroxyphenyl)-2,3-dimethyl-3,4,6,7-naphthalenetetrol. In the nuclear Overhauser effect spectroscopy (NOESY) spectrum of **4**, the NOE correlations between H-1 and Me-11, H-2 and H-4/Me-12/H-2′, and between H-4 and Me-12 are indicative of the 1β, 2α, 3α-hydroxy, and 4α relative configurations (Figure 3). The circular dichroism (CD) spectrum of **4** exhibited a positive Cotton effect at 244 and 275 nm, confirming the 1*R* absolute configuration [20]. The configurations at C-2, C-3, and C-4 were determined as 2*R*, 3*R*, and 4*S*, respectively. Thus, **4** was identified as (1*R*,2*R*,3*R*,4*S*)-1,2,4-trihydro-1-(3,4-dihydroxyphenyl)-2,3-dimethyl-3,4,6,7-naphthalenetetrol.

**Figure 4:**
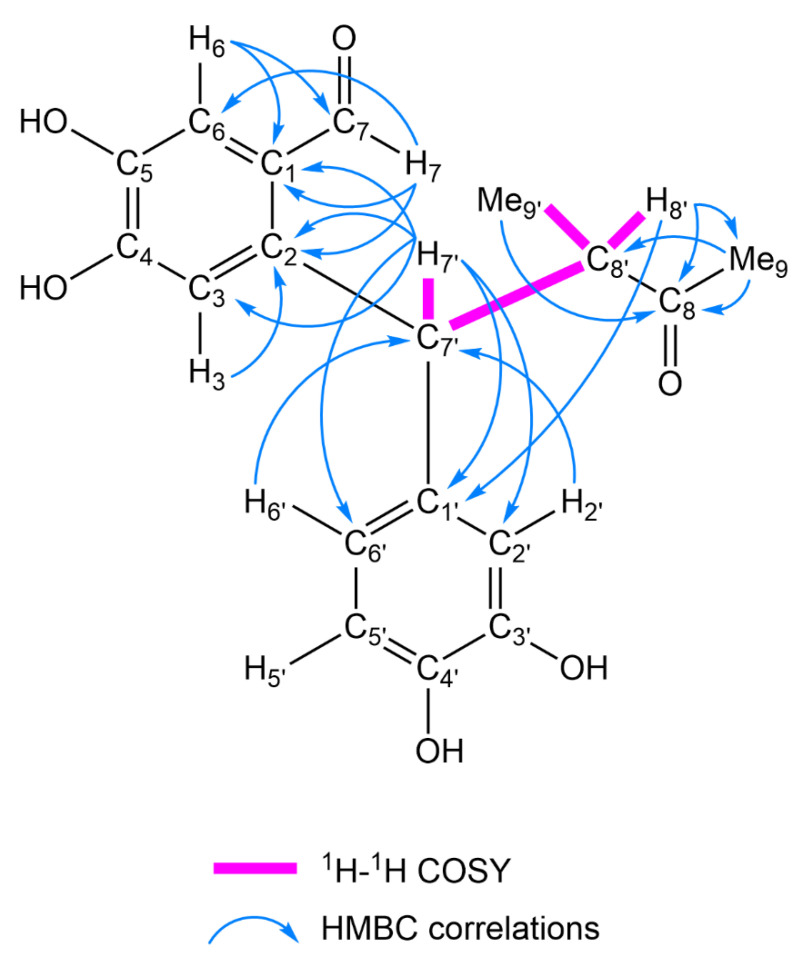
^1^H-^1^H COSY and key HMBC correlations of **5**.

Compound **5** (C_18_H_18_O_6_) was collected as an amorphous powder. The IR spectrum of **5** shows absorption bands of the hydroxy groups (3271 cm^−1^) and carbonyl groups (1715 and 1695 cm^−1^). The ^1^H- and ^13^C-NMR spectra of **5** showed signals of a 1,2,4,5-tetrasubstituted aromatic ring [δ_H_ 7.22 (1H, s, H-6), 7.03 (1H, s, H-3); δ_C_ 125.2, 140.3, 113.4, 151.1, 143.1, 116.4 (C-1–C-6)], a 1,2,4-trisubstituted aromatic ring [δ_H_ 6.78 (1H, d, *J* = 1.7 Hz, H-2′), 6.73 (1H, d, *J* = 8.0 Hz, H-5′), 6.70 (1H, dd, *J* = 8.0, 1.7 Hz, H-6′); δ_C_ 133.3, 114.6, 144.5, 143.1, 114.4, 118.9 (C-1′–C-6′)], two methyl groups [δ_H_ 2.09 (3H, s, Me-9), 1.08 (3H, d, *J* = 6.9 Hz, Me-9′); δ_C_ 27.1 (C-9), 15.1 (C-9′)], two methine groups [δ_H_ 5.05 (1H, d, *J* = 11.4 Hz, H-7′), 3.50 (1H, dq, *J* = 11.4, 6.9 Hz, H-8′); δ_C_ 51.1 (C-8′), 44.7 (C-7′)], an aldehyde group [δ_H_ 10.2 (1H, s, H-7); δ_C_ 190.4 (C-7)], and a carbonyl carbon [δ_C_ 213.2 (C-8)]. The spin-coupling correlation H-7′/H-8′/Me-9′ in the ^1^H-^1^H COSY spectrum of **5** indicates the existence of a structural fragment of -C_(7′)_H-C_(8′)_H(Me_(9′)_)- (Figure 4). The HMBC spectrum displayed long-range correlations from H-8′, Me-9′, and Me-9 to the C-8 carbonyl carbon. These correlations allowed the structural fragment to be extended to -C_(7′)_H-C_(8′)_H(Me_(9′)_)-C_(8)_(=O)-Me_(9)_, which was shown to be linked to C-1′ of the 1,2,4-trisubstituted aromatic ring and C-2 of the 1,2,4,5-tetrasubstituted aromatic ring based on the observed HMBC correlations between H-7′ and C-1′/C-2′/C-6′/C-1/C-2/C-3. Furthermore, the aldehyde group was confirmed to be attached to C-1 of the 1,2,4,5-tetrasubstituted aromatic ring based on the long-range correlation from the H-7 aldehyde proton to C-1/C-2/C-6 of the aromatic carbons (Figure 4). The configurations of C-7′ and C-8′ have yet to be determined. Accordingly, **5** was deduced to be 2-[1-(3,4-dihydroxyphenyl)-2-methyl-3-oxobutyl]-4,5-dihydroxybenzaldehyde.

Compound **6** (C_18_H_18_O_3_) was obtained as an amorphous powder and is suggested to be a benzofuran-type neolignan, the ^1^H- and ^13^C-NMR spectra of which resembled those of **12**, except for the signals arising from the aromatic ring bonded to C-2 of the benzofuran moiety. In the ^1^H-NMR spectrum of **6**, the aromatic proton showed a typical 1,2,4-trisubstituted spin-coupling system at δ_H_ 6.87 (1H, d, *J* = 2.1 Hz, H-2″), 6.80 (1H, d, *J* = 8.2 Hz, H-5″), and 6.76 (1H, dd, *J* = 8.2, 2.1 Hz, H-6″). Furthermore, the molecular formula of **6** was larger than that of **12** by an oxygen atom. The above data revealed that **6** is the C-3″ hydroxy derivative of **12**. Because the proton spin-coupling constant between H-2 and H-3 was 8.6 Hz, and the CD spectrum of **6** was in good agreement with that of **12** [17], the absolute configurations of C-2 and C-3 were determined to be *R* and *R*, respectively. Thus, **6** was identified as (2*R*,3*R*)-2,3-dihydro-2-(3,4-dihydroxyphenyl)-3-methyl-5-(*E*)-propenylbenzofuran.

The ^1^H- and ^13^C-NMR spectra of **7** (C_19_H_20_O_3_) were similar to those of **6**. However, the molecular formula of **7** was larger than that of **6** by CH_2_, and the presence of a methoxy group [δ_H_ 3.88 (3H, s); δ_C_ 54.6] was verified based on ^1^H- and ^13^C-NMR spectral analysis. Comparison of the ^13^C-NMR spectrum of **7** with that of **6** showed that the signal assignable to C-4″ was moved downfield by 2.5 ppm, whereas that attributable to C-5″ was shifted upfield by 3.5 ppm. Additionally, an HMBC correlation from δ_H_ 3.88 (OMe) to δ_C_ 147.2 (C-4″) was detected. The above data revealed that **7** is a C-4″-*O*-methyl derivative of **6**. Accordingly, **7** was deduced to be (2*R*,3*R*)-2,3-dihydro-2-(3-hydroxy-4-methoxyphenyl)-3-methyl-5-(*E*)-propenylbenzofuran.

### 2.2. Cytotoxicity of **1–17**

The cytotoxicity of **1**–**17** to HL-60 cells was evaluated using a modified 3-(4,5-dimethylthiazol-2-yl)-2,5-diphenyl-2*H*-tetrazolium bromide (MTT) assay (Table 1). Compounds **4**–**11** and **14**–**16** exhibited cytotoxicity to HL-60 cells, with IC_50_ values in the range of 2.7–17 μM. Comparison of the cytotoxicity of **8** with that of **1** and **2**, and the cytotoxicity of **9** with that of **3** suggests that glucosylation(s) of the aromatic group(s) diminished the cytotoxicity. In benzofuran-type neolignans (**6**, **7**, and **12**), the catechol group (C-3″, C-4″-hydroxy) was necessary to induce cytotoxicity (**6**), and C-4″-*O*-methylation reduced the cytotoxicity (**7**). For the tetrahydrofuran-type lignans (**13**–**17**), the presence of the catechol group was also essential for exerting cytotoxicity (**14**, **15**, and **16**).

### 2.3. Apoptosis Inducing Activity of **6**

**Figure 5:**
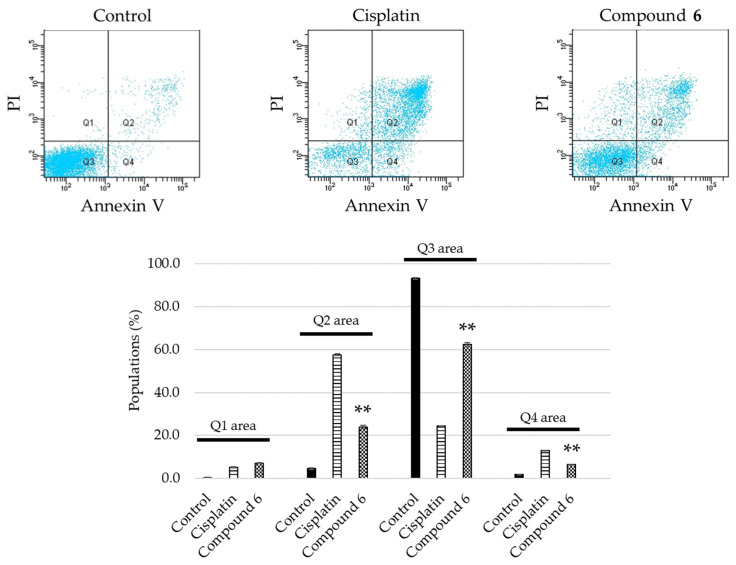
Detection of apoptosis in HL-60 cells treated with cisplatin or **6**. Top: HL-60 cells were treated with either 20 μM of cisplatin or 40 μM of **6** for 24 h, and then stained with Annexin V and propidium iodide (PI), followed by flow cytometry analysis. Bottom: the percentage of dead cells (Q1 area), late apoptotic cells (Q2 area), live cells (Q3 area), and early apoptotic cells (Q4 area) in the cell population are shown as mean ± S.E.M. of three experiments (** *p* < 0.001 vs. control group).

**Figure 6:**
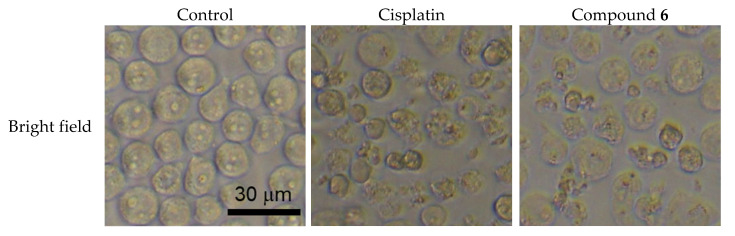

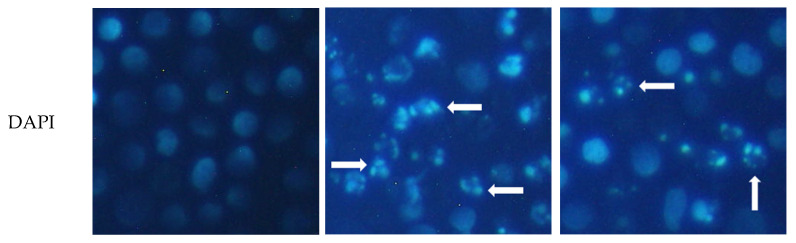
Morphology of HL-60 cells treated with cisplatin or **6**. HL-60 cells were stained with DAPI after treatment with either 20 μM of cisplatin or 40 μM of **6** for 24 h, and observed by a fluorescence microscopy (magnification: 200×).

**Figure 7:**
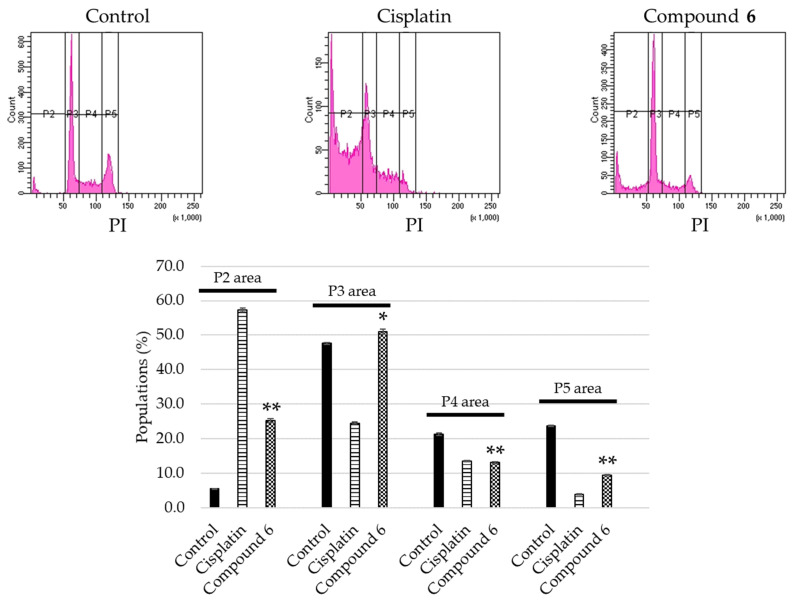
Cell cycle progression for HL-60 cells treated with cisplatin or **6**. Top: HL-60 cells were treated with either 20 μM of cisplatin or 40 μM of **6** for 24 h, and the cell cycle distribution was analyzed by flow cytometry. Bottom: percentage of cells in the sub-G_1_ (P2 area), G_0_/G_1_ (P3 area), S (P4 area), and G_2_/M (P5 area) phase are exhibited as mean ± S.E.M. of three experiments (** *p* < 0.001, * *p* < 0.05 vs. control group).

The apoptosis-inducing activity of **6** was evaluated. Among the unprecedented compounds, **6** exerted the highest cytotoxicity in HL-60 cells. HL-60 cells were treated with 40 μM of **6** for 24 h, stained with Annexin V and propidium iodide (PI), and analyzed by flow cytometry. The cell populations of early (Q4 area) and late (Q2 area) apoptotic cells, for which the vehicle control was 1.9 ± 0.033% and 4.5 ± 0.38%, increased to 6.5 ± 0.033% and 24 ± 0.87%, respectively (Figure 5). HL-60 cells treated with 40 μM of **6** were stained with 4′,6-diamidino-2-phenylindole (DAPI), and their morphology was observed under a fluorescence microscope. As shown in Figure 6, the HL-60 cells exhibited chromatin condensation and nuclear disassembly, which are representative phenomena in apoptosis. In addition, the cell cycle distribution of HL-60 cells was analyzed using a flow cytometer. When the cells were treated with 40 μM of **6** for 24 h, the sub-G1 population increased to 25 ± 0.58%, compared to 5.5 ± 0.088% in the vehicle control populations (Figure 7). Notably, the results showed that, for HL-60 cells treated with **6**, the population of cells in the G_0_/G_1_ phase (P3 area) increased (control: 48 ± 0.35%; **6**: 51 ± 0.71%), whereas those in the S phase declined (P4 area) (control: 21 ± 0.38%; **6**: 13 ± 0.29%) compared with the vehicle control.

## 3. Material and Methods

### 3.1. General Experimental Procedures

Optical rotations were measured on a JASCO P-1030 automatic digital polarimeter (Tokyo, Japan). IR, UV, and CD spectral data were collected using a FT-IR 620 spectrometer (JASCO), V-630 spectrometer (JASCO), and J-720 spectrometer (JASCO), respectively. NMR spectral data were obtained by a Bruker DPX-400 (^1^H-NMR: 400 MHz; ^13^C-NMR: 100 MHz), Bruker DRX-500 (^1^H-NMR: 500 MHz; ^13^C-NMR: 125 MHz), Bruker AV-500 (^1^H-NMR: 500 MHz; ^13^C-NMR: 125 MHz), or Bruker AV-600 (^1^H-NMR: 600 MHz; ^13^C-NMR: 150 MHz) spectrometer using a standard Bruker pulse programs at 300 K (Karlsruhe, Germany). Chemical shifts are rendered as δ values with reference to tetramethylsilane (TMS) as an internal standard. HR-ESI-TOF mass were measured using a Waters Micromass LCT mass spectrometer (Milford, MA, USA). Diaion HP-20 porous polymer polystyrene resin (Mitsubishi-Chemical, Tokyo, Japan), silica gel Chromatorex BW-300 (Fuji-Silysia Chemical, Aichi, Japan), and ODS silica gel COSMOSIL 75C_18_-OPN (Nacalai Tesque, Kyoto, Japan) were employed for CC. Thin layer chromatography (TLC) analysis carried out by precoated silica gel 60F_254_ or RP18 F_254_S plates (0.25 mm thick; Merch, Darmstadt, Germany), and the sample spots were detected by spraying the TLC plates with H_2_SO_4_/H_2_O (1:9), followed by heating. The HPLC system was constituted with the following instrument; a Tosoh CCPM pump (Tokyo, Japan), a Shodex OR-2 detector (Showa-Denko, Tokyo, Japan), a Rheodyne^TM^ injection port (Thermo Fisher Scientific, Waltham, MA, USA), and a Capcell Pak NH_2_ UG80 (4.6 mm i.d. × 250 mm, 5 μm; Shiseido, Tokyo, Japan). Enzymatic hydrolysis was conducted using β-D-glucosidase (Sigma, St. Louis, MO, USA). The following materials and biochemical-grade reagents were used for the cell culture assays: a 96-well flat-bottom plate (Iwaki Glass, Chiba, Japan); RPMI-1640 medium, cisplatin, and MTT (Sigma); penicillin G sodium salt and streptomycin sulfate (Gibco, Gland Island, NY, USA); paraformaldehyde and phosphate-buffered saline (PBS) (FUJIFILM Wako Pure Chemical Corporation, Osaka, Japan); fetal bovine serum (FBS) (Bio-Whittaker, Walkersville, MD, USA); a Spectra Classic microplate reader (Tecan, Salzburg, Austria).

### 3.2. Plant Material

The aerial parts of *L. tridentata* were purchased from Richters Herbs (Ontario, Canada) in 2007. A voucher specimen was kept at the herbarium of the Tokyo University of Pharmacy and Life Sciences.

### 3.3. Extraction and Isolation Procedures

The aerial parts of *L. tridentata* (dry weight, 3.0 kg) were extracted with hot MeOH (15 L × 4 times), and the extract was evaporated under reduced pressure. The MeOH extract (940 g) was loaded on a Diaion HP-20 column and successively eluted with MeOH/H_2_O (3:7), MeOH/H_2_O (1:1), MeOH, EtOH, and EtOAc. The MeOH eluted portion [Fraction (Fr) C; 477 g] was subjected to silica gel CC and eluted with CHCl_3_/MeOH/H_2_O (50:1:0; 19:1:0; 9:1:0; 40:10:1) to obtain 7 fractions (Frs. C-1–C-7). Fr. C-3 was separated by silica gel CC eluted with CHCl_3_/MeOH (19:1; 9:1) and ODS silica gel CC eluted with MeOH/H_2_O (1:3; 3:2; 2:1; 4:1) to obtain **8** (79 mg) and **13** (5.2 mg). Fr. C-4 was applied to silica gel CC eluted with CHCl_3_/MeOH/H_2_O (9:1:0; 40:10:1), and ODS silica gel CC eluted with MeOH/H_2_O (2:5; 1:2; 2:3; 1:1; 3:2; 2:1) and MeCN/H_2_O (2:3) to collect **4** (4.6 mg) and **5** (5.0 mg). Fr. C-5 was divided by silica gel CC eluted with CHCl_3_/MeOH/H_2_O (9:1:0; 50:10:1; 40:10:1), and ODS silica gel CC eluted with MeOH/H_2_O (4:3; 8:3) and MeCN/H_2_O (2:5; 1:1) to get **1** (11 mg). Fr. C-6 was subjected to silica gel CC eluted with CHCl_3_/MeOH/H_2_O (7:4:1; 27:18:5; 2:4:1; 60:10:1; 30:10:1), and ODS silica gel CC eluted with MeOH/H_2_O (1:2; 2:3; 9:11; 11:9; 2:1; 4:1) and MeCN/H_2_O (1:4; 1:3) to afford **2** (12 mg) and **3** (7.0 mg). The EtOH eluted portion (Fr. D; 41 g) was chromatographed on silica gel CC eluted with CHCl_3_/MeOH (19:1; 9:1) to get 8 fractions (Frs. D-1–D-8). Fr. D-2 was purified using silica gel CC eluted with hexane/EtOAc (5:1) and ODS silica gel CC eluted with MeOH/H_2_O (7:3; 4:1) to yield **7** (5.3 mg). Fr. D-5 was separated by silica gel CC eluted with CHCl_3_/MeOH (19;1) and hexane/EtOAc (3:1; 5:1) and ODS silica gel CC eluted with MeCN/H_2_O (1:1) to obtained **6** (12 mg) and **12** (4.7 mg). Fr. D-7 was loaded on silica gel CC eluted with hexane/EtOAc (2:1), hexane/CHCl_3_/MeOH (1:19:1; 4:38:1), and CHCl_3_/MeOH (19:1), and ODS silica gel CC eluted with MeOH/H_2_O (3:2) and MeCN/H_2_O (1:2) to afford **10** (7.8 mg), **11** (2.6 mg), **16** (10 mg), and **17** (12 mg). Fr. D-8 was divided by silica gel CC eluted with CHCl_3_/MeOH (19:1; 9:1) to furnish **9** (17 mg), **14** (12 mg), and **15** (21 mg).

### 3.4. Structural Elucidation

Compound **1**: Amorphous powder; [α]_D_^25^ −71.6 (*c* = 0.09, MeOH); IR (film) ν_max_: 3365 (OH), 1613, 1513, 1453 (aromatic ring) cm^−1^; HR-ESI-TOF-MS *m/z*: 469.1843 [M + Na]^+^ (calculated for C_24_H_30_NaO_8_: 469.1838); UV λ_max_ (MeOH): 281 (log ε = 3.74) nm; CD λ_max_ (MeOH) (Δε): 232 (−9.41), 273 (−1.32), 289 (+1.97). ^1^H-NMR spectral data for the aglycone moiety (500 MHz, CD_3_OD): δ_H_ 6.97 (1H, s, H-5), 6.85 (2H, d, *J* = 8.5 Hz, H-2′ and H-6′), 6.71 (2H, d, *J* = 8.5 Hz, H-3′ and H-5′), 6.31 (1H, s, H-8), 3.60 (1H, d, *J* = 6.6 Hz, H-1), 2.90 (1H, dd, *J* = 16.0, 5.5 Hz, H-4a), 2.49 (1H, dd, *J* = 16.0, 7.0 Hz, H-4b), 2.04 (1H, m, H-3), 1.94 (1H, ddd, *J* = 6.8, 6.6, 2.5 Hz, H-2), 0.93 (3H, d, *J* = 7.0 Hz, Me-12), 0.91 (3H, d, *J* = 6.8 Hz, Me-11). For ^1^H-NMR spectral data of the sugar moiety, see Table 2. For ^13^C-NMR spectral data, see Table 3. For NMR data, see Supplementary Materials.

Compound **2**: Amorphous powder; [α]_D_^25^ −99.8 (*c* = 0.10, MeOH); IR (film) ν_max_: 3363 (OH), 1643, 1507, 1453 (aromatic ring) cm^−1^; HR-ESI-TOF-MS *m/z*: 631.2376 [M + Na]^+^ (calculated for C_30_H_40_NaO_13_: 631.2367); UV λ_max_ (MeOH): 282 (log ε = 3.78) nm; CD λ_max_ (MeOH) (Δε): 237 (−10.2), 270 (−2.66), 286 (+1.92). ^1^H-NMR spectral data for the aglycone moiety (500 MHz, CD_3_OD): δ_H_ 7.03 (2H, d, *J* = 8.7 Hz, H-3′ and H-5′), 6.98 (2H, d, *J* = 8.7 Hz, H-2′ and H-6′), 6.96 (1H, s, H-5), 6.28 (1H, s, H-8), 3.67 (1H, d, *J* = 6.6 Hz, H-1), 2.91 (1H, dd, *J* = 16.4, 5.3 Hz, H-4a), 2.51 (1H, dd, *J* = 16.4, 7.1 Hz, H-4b), 2.04 (1H, m, H-3), 1.95 (1H, ddd, *J* = 6.9, 6.6, 2.9 Hz, H-2), 0.93 (3H, d, *J* = 6.9 Hz, Me-12), 0.92 (3H, d, *J* = 6.9 Hz, Me-11). For ^1^H-NMR spectral data of the sugar moieties, see Table 2. For ^13^C-NMR spectral data, see Table 3. For NMR data, see Supplementary Materials.

Compound **3**: Amorphous powder; [α]_D_^25^ −104.5 (*c* = 0.10, MeOH); IR (film) ν_max_: 3287 (OH), 1639, 1506, 1453 (aromatic ring) cm^−1^; HR-ESI-TOF-MS *m/z*: 809.2808 [M + Na]^+^ (calculated for C_36_H_50_NaO_19_: 809.2844); UV λ_max_ (MeOH): 281 (log ε = 3.73) nm; CD λ_max_ (MeOH) (Δε): 233 (−11.2), 270 (−3.23), 286 (+1.68). ^1^H-NMR spectral data for the aglycone moiety (600 MHz, C_5_D_5_N): δ_H_ 7.54 (1H, d, *J* = 8.3 Hz, H-5′), 7.51 (1H, br s, H-2′), 7.38 (1H, s, H-5), 6.85 (1H, s, H-8), 6.68 (1H, d, *J* = 8.3 Hz, H-6′), 3.76 (1H, d, *J* = 5.2 Hz, H-1), 2.70 (1H, dd, *J* = 16.4, 4.8 Hz, H-4a), 2.31 (1H, dd, *J* = 16.4, 7.9 Hz, H-4b), 1.92 (1H, m, H-3), 1.91 (1H, m, H-2), 0.79 (3H, d, *J* = 6.7 Hz, Me-11), 0.69 (3H, d, *J* = 6.7 Hz, Me-12). For ^1^H-NMR spectral data of the sugar moieties, see Table 2. For ^13^C-NMR spectral data, see Table 3. For NMR data, see Supplementary Materials.

Compound **4**: Amorphous powder; [α]_D_^25^ 36.8 (*c* = 0.10, MeOH); IR (film) ν_max_: 3348 (OH), 1607, 1521, 1448 (aromatic ring) cm^−1^; HR-ESI-TOF-MS *m/z*: 355.1118 [M + Na]^+^ (calculated for C_18_H_20_NaO_6_: 355.1158); UV λ_max_ (MeOH): 285 (log ε = 3.75) nm; CD λ_max_ (MeOH) (Δε): 244 (+1.13), 275 (+4.64), 294 (−5.53). ^1^H-NMR spectral data (400 MHz, CD_3_OD): δ_H_ 7.04 (1H, s, H-5), 6.73 (1H, d, *J* = 8.0 Hz, H-5′), 6.54 (1H, d, *J* = 2.0 Hz, H-2′), 6.51 (1H, dd, *J* = 8.0, 2.0 Hz, H-6′), 6.15 (1H, s, H-8), 4.48 (1H, s, H-4), 3.56 (1H, d, *J* = 11.0 Hz, H-1), 1.88 (1H, dd, *J* = 11.0, 6.7 Hz, H-2), 1.41 (3H, s, Me-12), 0.92 (3H, d, *J* = 6.7 Hz, Me-11). For ^13^C-NMR spectral data, see Table 3. For NMR data, see Supplementary Materials.

Compound **5**: Amorphous powder; [α]_D_^25^ −87.0 (*c* = 0.10, MeOH); IR (film) ν_max_: 3271 (OH), 1715 and 1695 (C=O), 1667, 1519, 1453 (aromatic ring) cm^−1^; HR-ESI-TOF-MS *m/z*: 353.1021 [M + Na]^+^ (calculated for C_18_H_18_NaO_6_: 353.1001); UV λ_max_ (MeOH): 282 (log ε = 3.97) nm; CD λ_max_ (MeOH) (Δε): 238 (+11.6), 319 (−3.72). ^1^H-NMR spectral data (400 MHz, CD_3_OD): δ_H_ 10.2 (1H, s, H-7), 7.22 (1H, s, H-6), 7.03 (1H, s, H-3), 6.78 (1H, d, *J* = 1.7 Hz, H-2′), 6.73 (1H, d, *J* = 8.0 Hz, H-5′), 6.70 (1H, dd, *J* = 8.0, 1.7 Hz, H-6′), 5.05 (1H, d, *J* = 11.4 Hz, H-7′), 3.50 (1H, dq, *J* = 11.4, 6.9 Hz, H-8′), 2.09 (3H, s, Me-9), 1.08 (3H, d, *J* = 6.9 Hz, Me-9′). For ^13^C-NMR spectral data, see Table 3. For NMR data, see Supplementary Materials.

Compound **6**: Amorphous powder; [α]_D_^25^ 95.6 (*c* = 0.10, MeOH); IR (film) ν_max_: 3232 (OH), 1609, 1524, 1486 (aromatic ring) cm^−1^; HR-ESI-TOF-MS *m/z*: 283.1386 [M + H]^+^ (calculated for C_18_H_19_O_3_: 283.1334); UV λ_max_ (MeOH): 264 (log ε = 4.37) nm; CD λ_max_ (MeOH) (Δε): 230 (−3.44), 262 (+12.0), 269 (+10.2). ^1^H-NMR spectral data (500 MHz, CD_3_OD): δ_H_ 7.20 (1H, br s, H-4), 7.13 (1H, dd, *J* = 8.2, 1.6 Hz, H-6), 6.87 (1H, d, *J* = 2.1 Hz, H-2″), 6.80 (1H, d, *J* = 8.2 Hz, H-5″), 6.76 (1H, dd, *J* = 8.2, 2.1 Hz, H-6″), 6.71 (1H, d, *J* = 8.2 Hz, H-7), 6.39 (1H, dd, *J* = 15.8, 1.7 Hz, H-1′), 6.13 (1H, dq, *J* = 15.8, 6.6 Hz, H-2′), 5.01 (1H, d, *J* = 8.6 Hz, H-2), 3.35 (1H, m, H-3), 1.88 (3H, dd, *J* = 6.6, 1.7 Hz, Me-3′), 1.40 (3H, d, *J* = 6.8 Hz, Me-10). For ^13^C-NMR spectral data, see Table 3. For NMR data, see Supplementary Materials.

Compound **7**: Amorphous powder; [α]_D_^25^ 113.1 (*c* = 0.10, MeOH); IR (film) ν_max_: 3366 (OH), 1650, 1512, 1484 (aromatic ring) cm^−1^; HR-ESI-TOF-MS *m/z*: 297.1489 [M + H]^+^ (calculated for C_19_H_21_O_3_: 297.1491); UV λ_max_ (MeOH): 265 (log ε = 4.62) nm; CD λ_max_ (MeOH) (Δε): 221 (−14.5), 261 (+25.0), 271 (+21.4). ^1^H-NMR spectral data (500 MHz, CD_3_OD): δ_H_ 7.20 (1H, br s, H-4), 7.14 (1H, br d, *J* = 8.2 Hz, H-6), 6.94 (1H, d, *J* = 8.0 Hz, H-5″), 6.89 (1H, d, *J* = 2.0 Hz, H-2″), 6.87 (1H, dd, *J* = 8.0, 2.0 Hz, H-6″), 6.72 (1H, d, *J* = 8.2 Hz, H-7), 6.39 (1H, dd, *J* = 15.7, 1.6 Hz, H-1′), 6.13 (1H, dq, *J* = 15.7, 6.6 Hz, H-2′), 5.04 (1H, d, *J* = 8.5 Hz, H-2), 3.88 (3H, s, OMe), 3.35 (1H, m, H-3), 1.87 (3H, dd, *J* = 6.6, 1.6 Hz, H-3′), 1.40 (3H, d, *J* = 6.8 Hz, Me-10). For ^13^C-NMR spectral data, see Table 3. For NMR data, see Supplementary Materials.

Enzymatic hydrolysis of **1**–**3**: Compounds **1** (4.0 mg), **2** (5.1 mg), and **3** (2.7 mg) were independently dealt with β-D-glucosidase (each 30 mg) in AcOH/AcONa (pH 5.0, each 5.0 mL) at 28 °C for 42 h, 16 h, and 28 h, respectively. Each reactant was divided by silica gel CC eluted with CHCl_3_/MeOH/H_2_O (7:4:1) to afford **8** (1.4 mg) from **1**, **8** (1.8 mg) from **2**, **9** (0.1 mg) from **3**, and sugar fractions (1.1 mg from **1**, 1.3 mg from **2**, and 1.3 mg from **3**), respectively. HPLC analysis of D-glucose were carried out following conditions: solvent, MeCN/H_2_O (17:3); flow rate, 1.0 mL/min. D-Glucose was specified by comparing the retention time and optical rotation with those of an authentic sample: D-glucose (16.45, positive optical rotation).

### 3.5. Assay for Cytotoxic Activity

HL-60 cells (JCRB 0085; Human Science Research Resources Bank, Osaka, Japan) were maintained in RPMI-1640 medium, which contained heat-inactivated 10% FBS supplemented with L-glutamine, penicillin G sodium salt, and 100 μg/mL streptomycin sulfate. The cells kept in a humidified incubator at 37 °C with 5% CO_2_. HL-60 cells (4 × 10^4^ cells/mL) seeded in a 96-well plate. After 24 h, 4 μL of an EtOH/H_2_O (1:1) solution containing the test samples was added and 4 μL of EtOH/H_2_O (1:1) was added into control wells. HL-60 cells treated with each compound for 72 h. Then, cell viability was measured with a modified MTT reduction assay method [21]. Briefly, 10 μL of MTT solution (5 mg/mL in PBS) was added into each well, and then incubated at 37 °C. After 4 h, dissolved the formazan in dimethyl sulfoxide (DMSO) and measured the absorbance at a wavelength of 405 nm. A dose–response curve was plotted for **4**–**11** and **14**–**16**, all of which resulted in less than 50% cell growth at a concentration of 20 μM, and the exact concentration at which 50% inhibition (IC_50_) of cell growth occurred was calculated.

### 3.6. Detection of Apoptosis

HL-60 cells (1 × 10^6^ cells/well) were pre-incubated for 4 h in a 6 well plate, and then treated with either EtOH/H_2_O (1:1) (control), 20 μM of cisplatin, or 40 μM of **6**. After 24 h treatment, HL-60 cells were collected and washed with PBS, and incubated for 15 min at 28 °C in 1 × Annexin V binding buffer containing Annexin V-FITC and propidium iodide (PI) as provided by the manufacturer (15342, Nacalai Tesque). Apoptotic cells were analyzed by a BD FACSCelesta^TM^ flow cytometer (BD Biosciences, Franklin Lakes, NJ, USA).

### 3.7. DAPI Staining

HL-60 cells (5 × 10^5^ cells/mL) were pre-incubated in a 96 well plate. After 24 h, HL-60 cells were delt with either EtOH/H_2_O (1:1), 20 μM of cisplatin, or 40 μM of **6** for 24 h. After treatment, the cells fixed with 1% glutaraldehyde for 30 min at 28 °C. Then stained with DAPI (0.5 μg/mL in PBS) for 10 min at 28 °C. Finally, HL-60 cells were observed by a CKX41 fluorescence microscopy (Olympus, Tokyo, Japan) [22].

### 3.8. Cell Cycle Distribution Analysis

HL-60 cells (1 × 10^6^ cells/well) were seeded for 4 h in a 6 well plate, and then treated with either EtOH/H_2_O (1:1), 20 μM of cisplatin, or 40 μM of **6**. HL-60 cells were collected using PBS and fixed EtOH/H_2_O (7:3) overnight at −20 °C. Following procedures were carried out same as previously described [23]. Analysis of cell cycle distribution was performed by a BD FACSCelesta^TM^ flow cytometer (BD Biosicences).

### 3.9. Statistical Anlysis

Statistical analysis was carried out one-way analysis of variance (ANOVA) followed by Dunnett′s test. A probability (*p*) value of less than 0.001 or 0.05 was considered to represent a statistically significant difference.

## 4. Conclusions

A phytochemical investigation of the aerial parts of *L. tridentata* was performed to obtain 17 lignans and lignan glycosides (**1**–**17**), including seven novel compounds (**1**–**7**). Compound **3** has a unique structure with the D-glucosyl moiety on three different aromatic hydroxy groups. Compounds **4**–**11** and **14**–**16** are cytotoxic to HL-60 cells, with IC_50_ values in the range of 2.7–17 μM. Additionally, **6** induced apoptotic cell death in HL-60 cells. The apoptosis-inducing mechanism of **6** is currently under investigation.

## Figures and Tables

**Table 1:** Cytotoxic activity of **1**–**17** and cisplatin for HL-60 cells ^(^^1)^.

Compounds	IC_50_ (μM)			Compounds	IC_50_ (μM)		
**1**	>20			**10**	4.2	±	0.25
**2**	>20			**11**	2.7	±	0.23
**3**	>20			**12**	>20		
**4**	16	±	0.95	**13**	>20		
**5**	17	±	0.83	**14**	5.1	±	0.14
**6**	4.1	±	0.52	**15**	16	±	1.5
**7**	15	±	1.9	**16**	9.3	±	0.45
**8**	9.2	±	0.44	**17**	>20		
**9**	11	±	0.17	Cisplatin	1.1	±	0.19

^(1)^ Data represent the mean value ± S.E.M. from three experiments performed.

**Table 2:** ^1^H-NMR spectral data for the sugar moieties of **1**–**3**.

1					2					3				
Positions		δ_H_		*J* (Hz)	Positions		δ_H_		*J* (Hz)	Positions		δ_H_		*J* (Hz)
Glc 1″		4.78	d	8.0	Glc 1″		4.78	d	7.9	Glc 1″		5.53	d	8.0
2″		3.52	dd	9.0, 8.0	2″		3.52	m		2″		4.34	m	
3″		3.51	dd	9.0, 8.5	3″		3.50	m		3″		4.28	m	
4″		3.45	dd	9.5, 8.5	4″		3.42	m		4″		4.36	m	
5″		3.46	m		5″		3.47	m		5″		4.10	m	
6″	a	3.96	dd	12.0, 2.0	6″	a	3.95	dd	12.1, 1.8	6″	a	4.57	br d	11.0
	b	3.76	dd	12.0, 5.0		b	3.76	dd	12.1, 5.4		b	4.42	br d	11.0
														
					Glc 1″′		4.92	d	7.9	Glc 1″′		5.62	d	7.5
					2″′		3.50	m		2″′		4.28	m	
					3″′		3.48	m		3″′		4.30	m	
					4″′		3.42	m		4″′		4.32	m	
					5″′		3.45	m		5″′		3.93	m	
					6″′	a	3.92	br d	12.2	6″′	a	4.44	br d	11.3
						b	3.72	dd	12.2, 4.6		b	4.38	br d	11.3
														
										Glc 1″″		5.56	d	7.4
										2″″		4.32	m	
										3″″		4.32	m	
										4″″		4.32	m	
										5″″		4.01	m	
										6″″	a	4.50	br d	10.7
											b	4.39	br d	10.7

The ^1^H-NMR spectra of **1** and **2** were recorded at 500 MHz in CD_3_OD and **3** was recorded at 600 MHz in C_5_D_5_N.

**Table 3:** ^13^C-NMR spectral data for **1**–**7**.

Positions	1	2	3	4	5	6	7
1	49.4	49.5	50.9	48.3	125.2	-	-
2	40.2	40.2	40.8	43.6	140.3	92.4	92.1
3	28.7	28.7	28.8	72.3	113.4	44.6	44.7
4	34.2	34.2	34.6	73.5	151.1	120.0	120.0
5	116.9	117.0	119.6	112.5	143.1	130.8	130.8
6	143.3	143.4	145.4	142.7	116.4	125.4	125.4
7	144.2	144.3	147.4	143.2	190.4	107.9	108.0
8	116.7	116.7	118.9	115.0	213.2	157.8	157.7
9	133.2	132.8	133.6	131.5	27.1	131.9	131.8
10	127.1	127.0	127.5	128.7		16.6	16.6
11	14.3	14.2	15.4	11.0			
12	14.1	14.1	16.4	21.9			
1′	137.4	140.5	143.0	137.6	133.3	130.3	130.3
2′	129.1	129.1	120.8	115.5	114.6	121.5	121.5
3′	113.9	115.5	148.3	144.4	144.5	16.5	16.6
4′	154.5	155.4	147.2	142.7	143.1		
5′	113.9	115.5	118.9	114.1	114.4		
6′	129.1	129.1	124.1	120.3	118.9		
7′					44.7		
8′					51.1		
9′					15.1		
1″	102.7	102.7	105.1			131.8	133.3
2″	73.1	73.1	75.2			112.3	112.1
3″	75.8	76.1	78.3			144.7	145.9
4″	69.5	69.5	71.2			144.7	147.2
5″	76.4	76.2	79.0			114.3	110.8
6″	60.6	60.6	62.3			117.1	116.8
OMe							54.6
1″′		100.5	103.8				
2″′		73.1	75.0				
3″′		75.8	78.3				
4″′		69.5	71.2				
5″′		76.4	78.5				
6″′		60.6	62.2				
1″″			104.1				
2″″			75.1				
3″″			78.1				
4″″			71.2				
5″″			78.8				
6″″			62.3				

The ^13^C-NMR of **4** and **5** were recorded at 100 MHz in CD_3_OD, **1**, **2**, **6**, and **7** at 125 MHz in CD_3_OD, and **3** at 150 MHz in C_5_D_5_N.

## Supplementary Materials

The following are available online. Figures S1–S36 exhibited NMR spectral data of **1****–****7**.

## Sample Availability

Isolated compounds are not available from the authors.
