# Supplementary material for: Structure and Cytotoxicity of Novel Lignans and Lignan Glycosides from the Aerial Parts of Larrea tridentata
Source: Molecules. 2021 Oct 14;26(20):6186. doi: 10.3390/molecules26206186 (PMC8540297; doi:10.3390/molecules26206186)
Supplement: Supplementary file 1 [file molecules-26-06186-s001.zip › molecules-1413868-supplementary.pdf]

Supplementary material

Structure and cytotoxicity of novel lignans and lignan glycosides from the aerial parts of *Larrea tridentata*

Akihito Yokosuka, Tomoki Iguchi*, Maki Jitsuno, and Yoshihiro Mimaki

School of Pharmacy, Tokyo University of Pharmacy and Life Sciences, 1432-1, Horinouchi, Hachioji, Tokyo 192-0392, Japan; yokosuka@toyaku.ac.jp (A.Y.), sasatatesatoru@gmail.com (M.J.), mimakiy@toyaku.ac.jp (Y.M.)

* Correspondence: iguchit@toyaku.ac.jp; Tel.: +81-42-676-4575

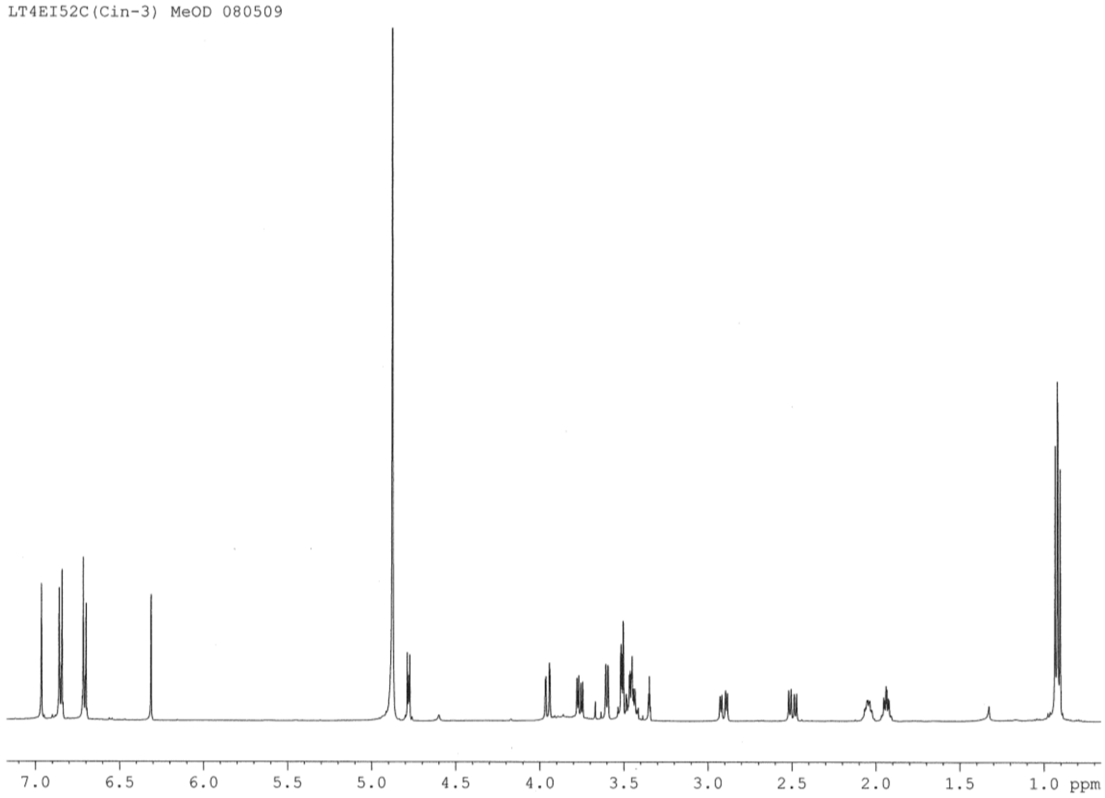

Figure S1. ^{1}H-NMR spectrum of **1**

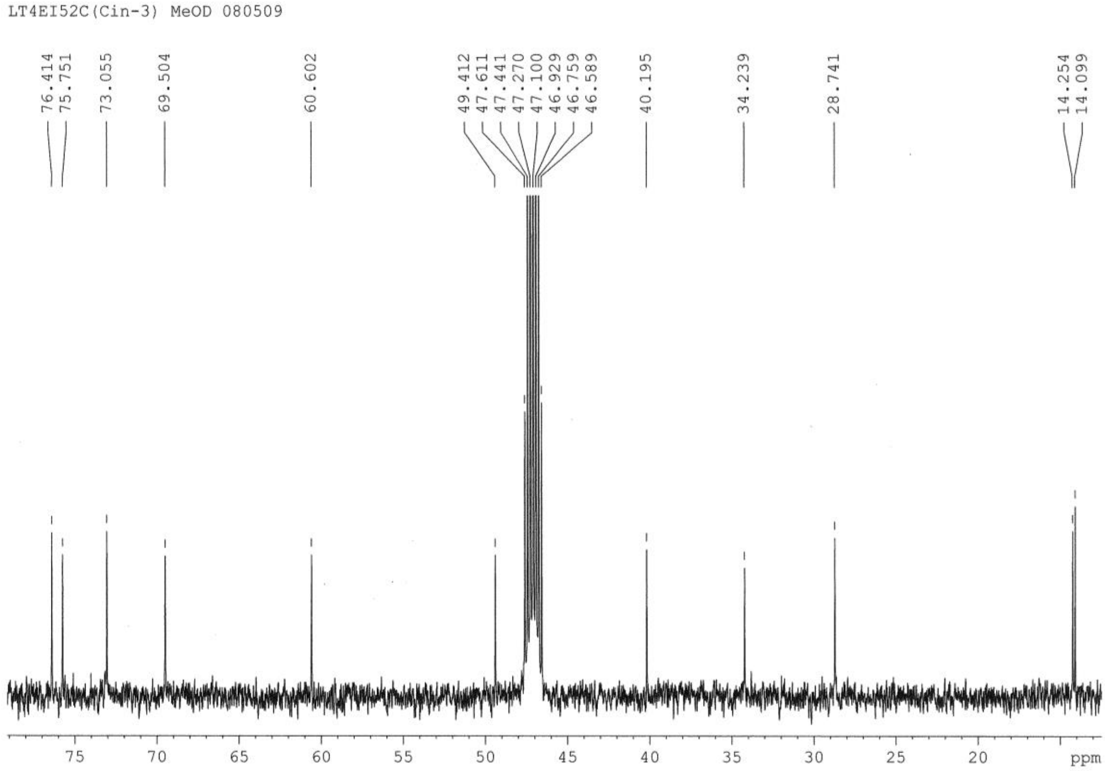

Figure S2-1. ^{13}C-NMR spectrum of **1**

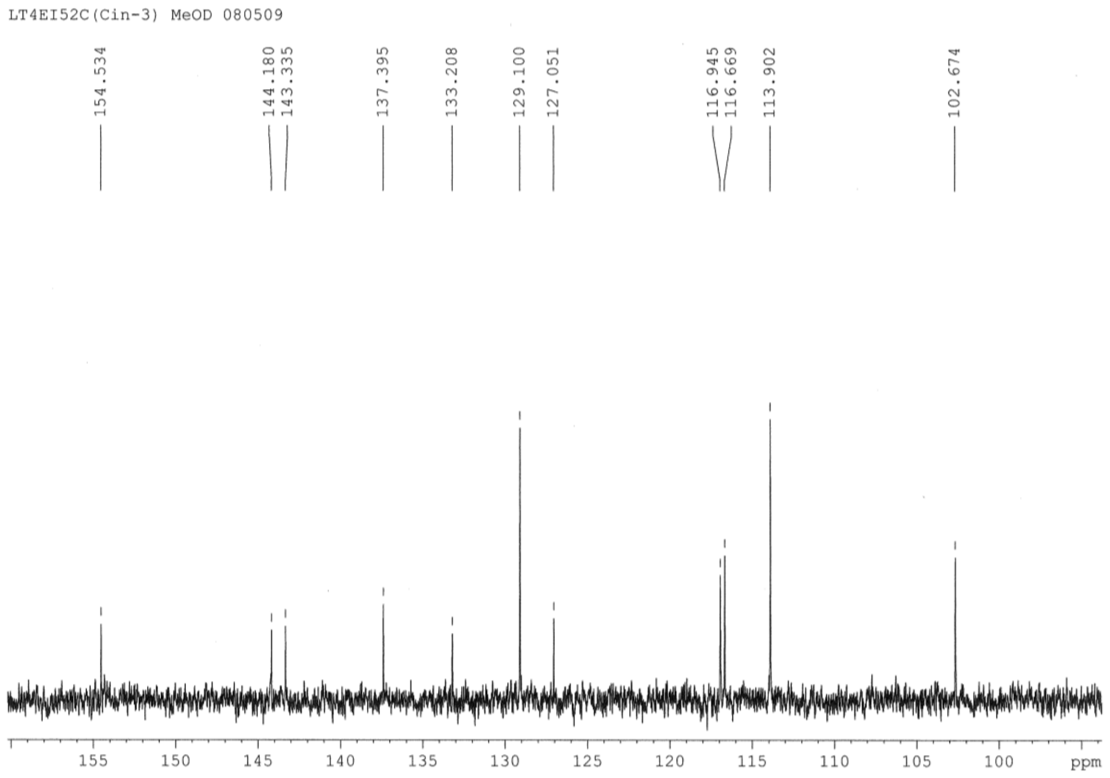

Figure S2-2. ^{13}C-NMR spectrum of **1**

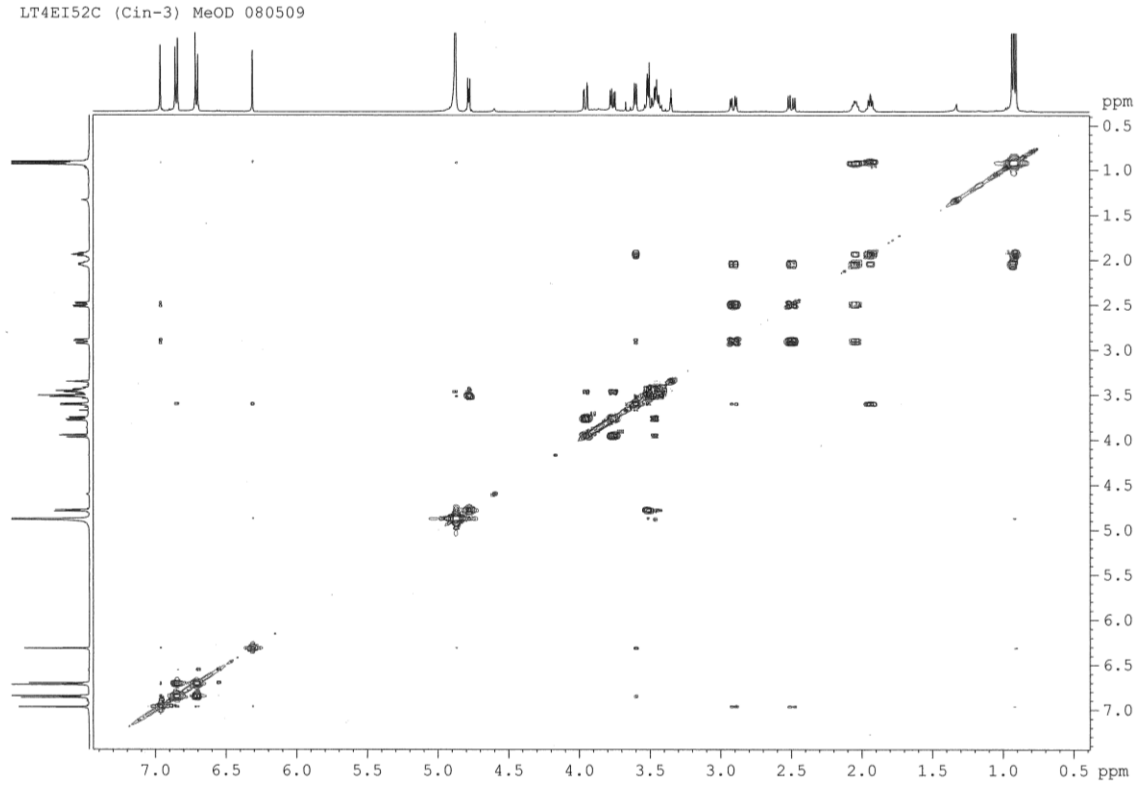

Figure S3. ^{1}H-^{1}H COSY of **1**

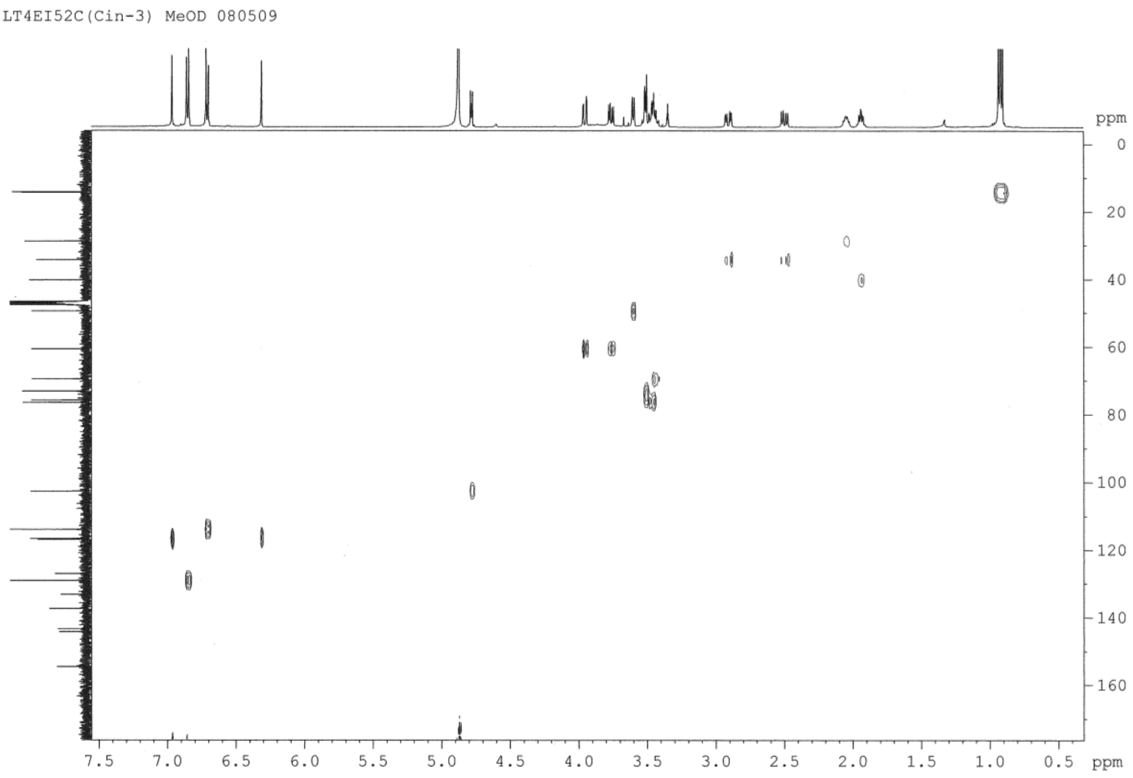

Figure S4. HSQC spectrum of **1**

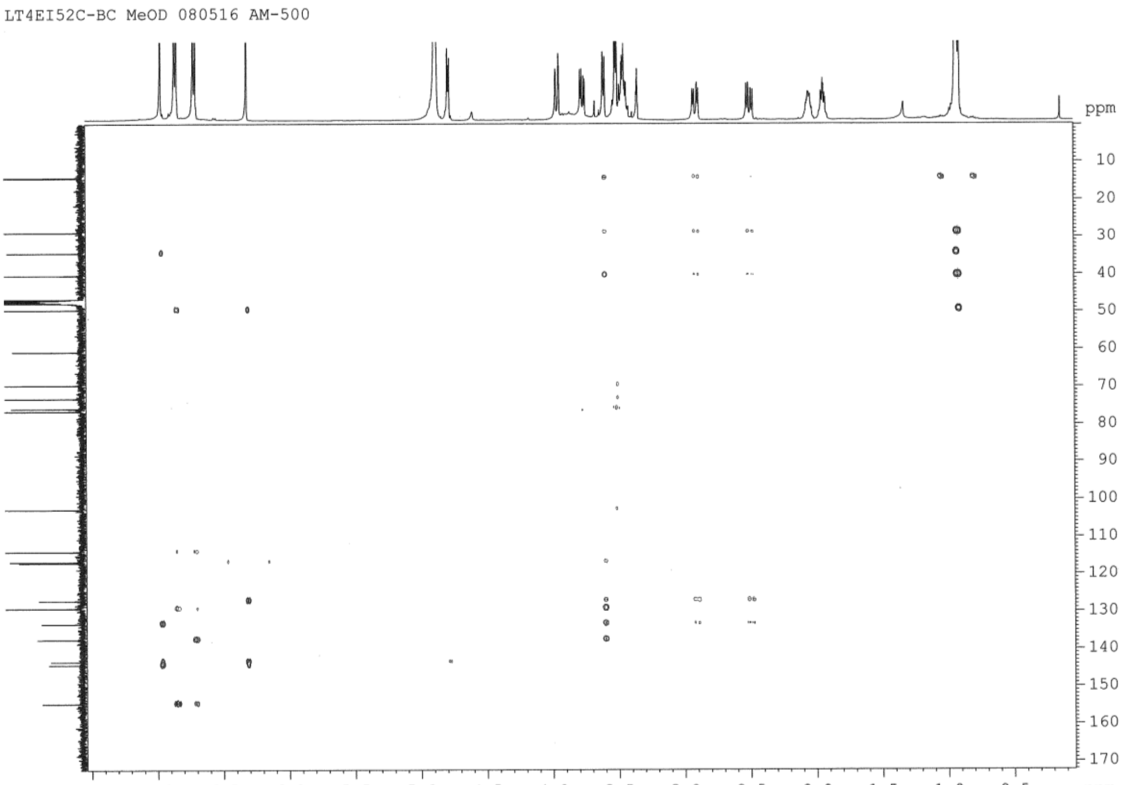

Figure S5. HMBC spectrum of **1**

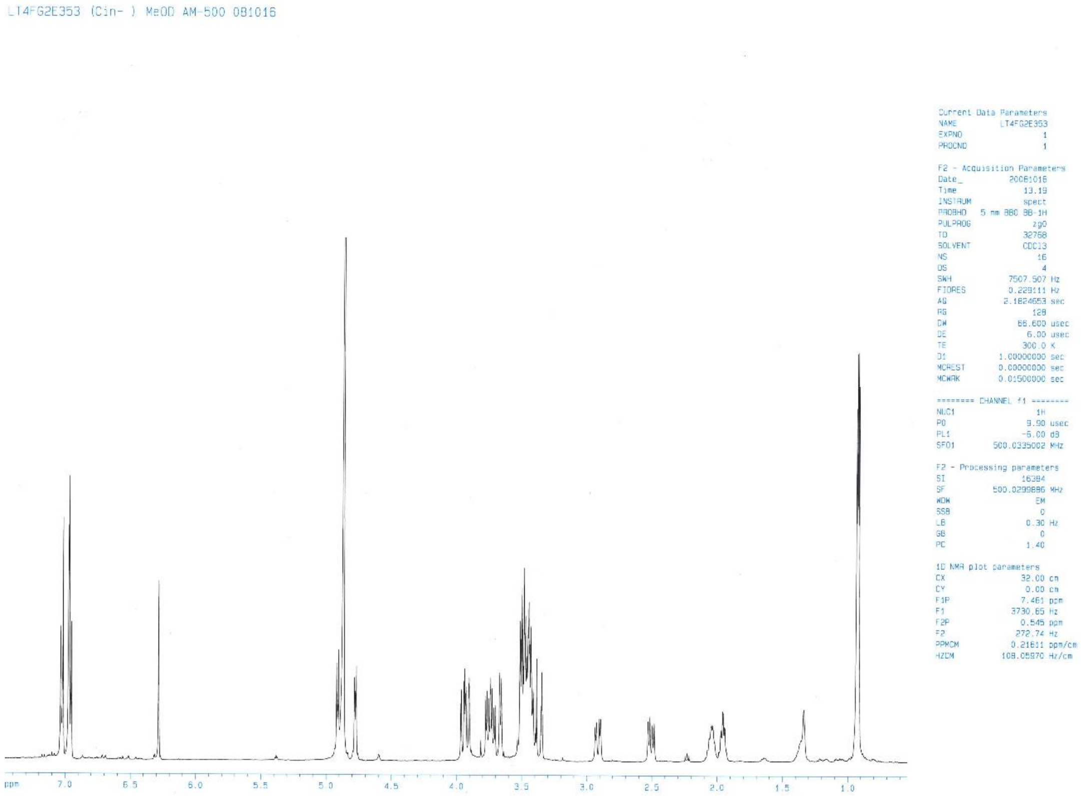

Figure S6. ^{1}H-NMR spectrum of **2**

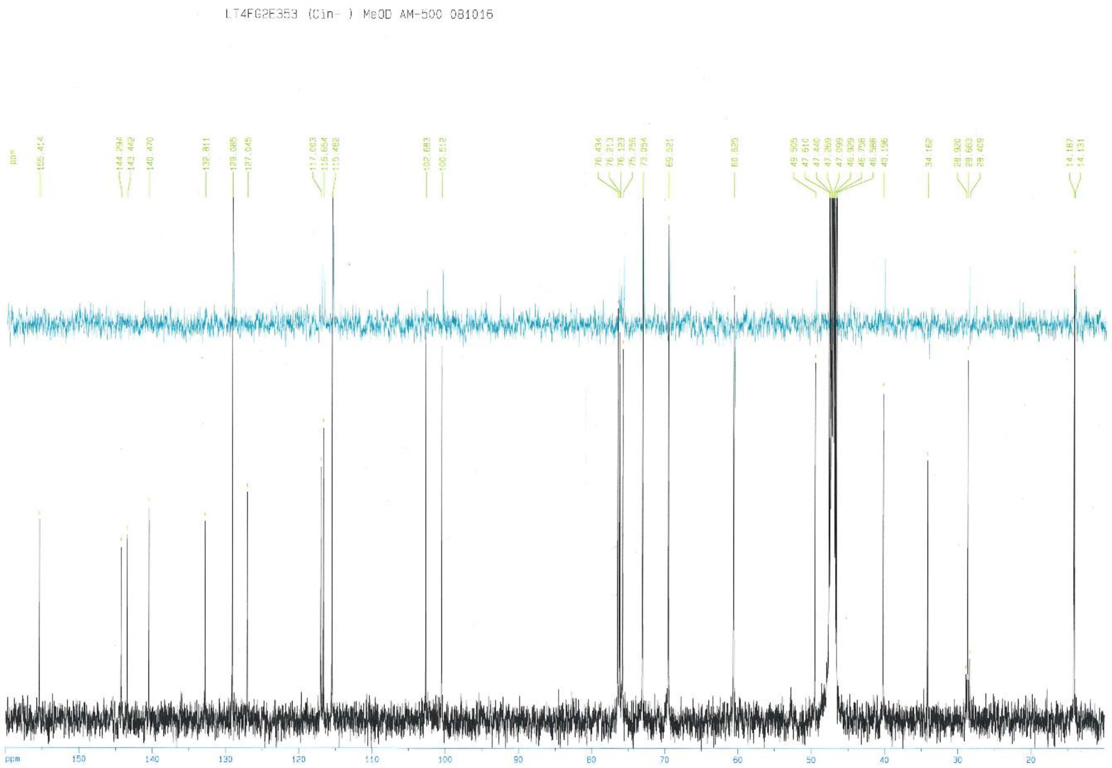

Figure S7. ^{13}C-NMR spectrum of **2**

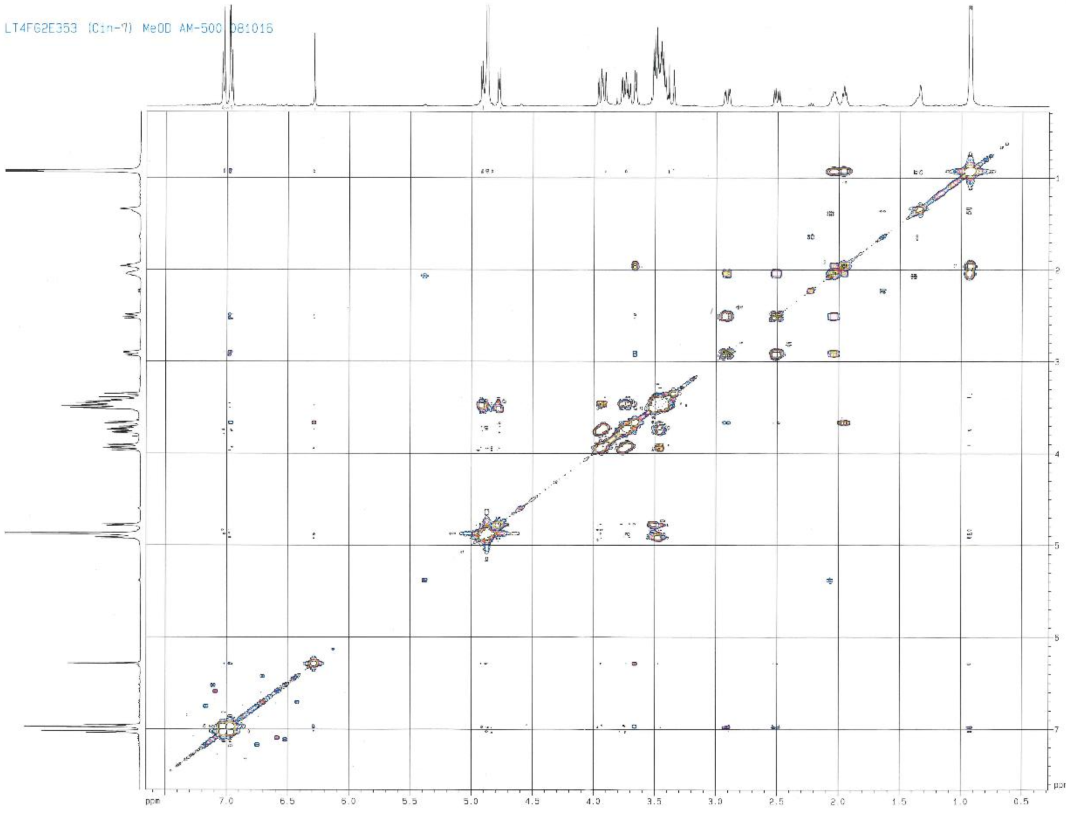

Figure S8. ^{1}H-^{1}H COSY of **2**

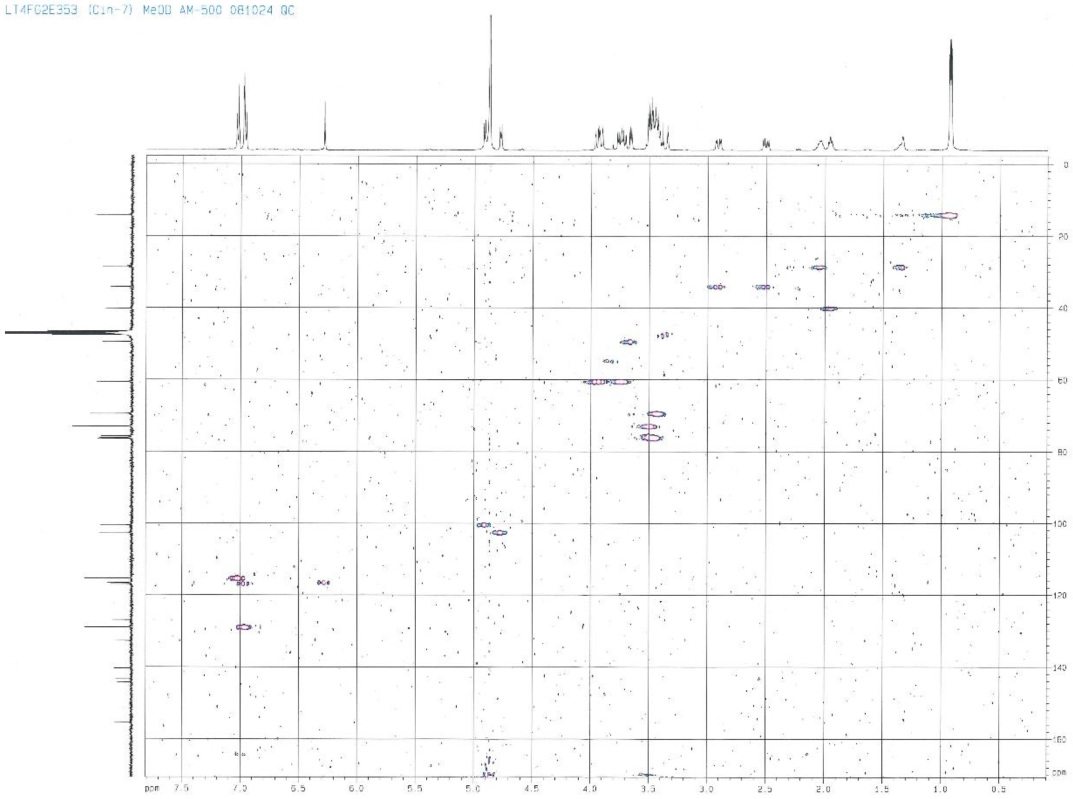

Figure S9. HSQC spectrum of **2**

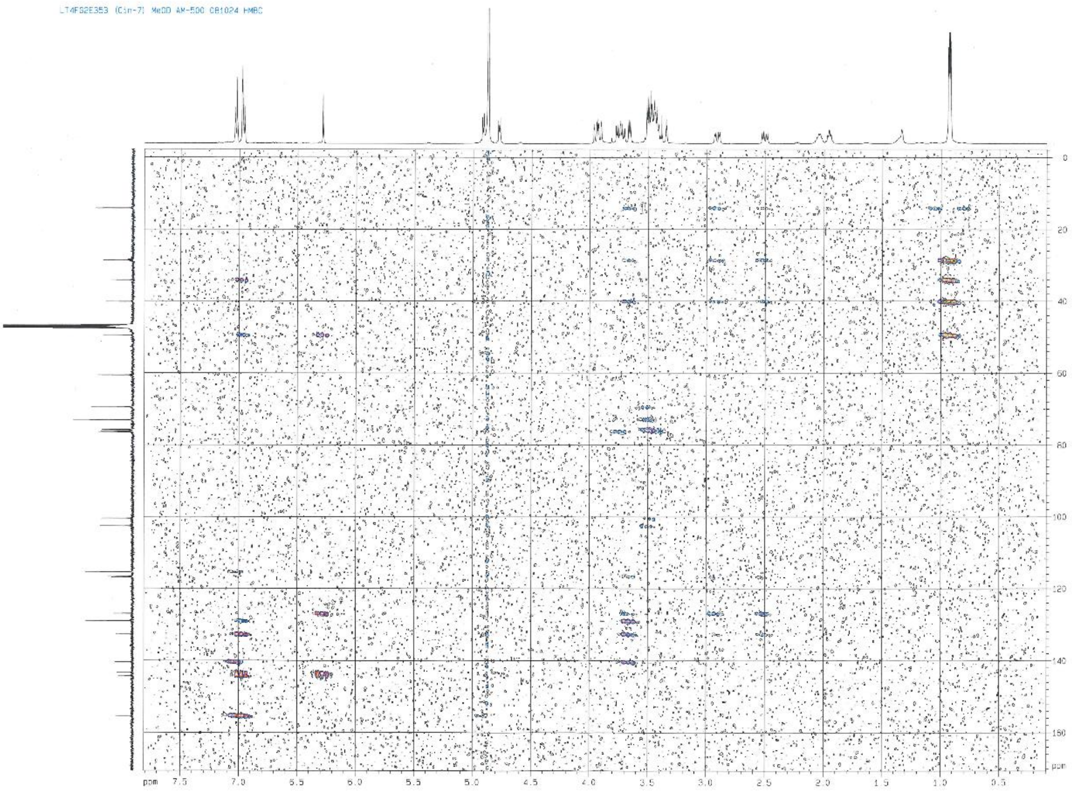

Figure S10. HMBC spectrum of **2**

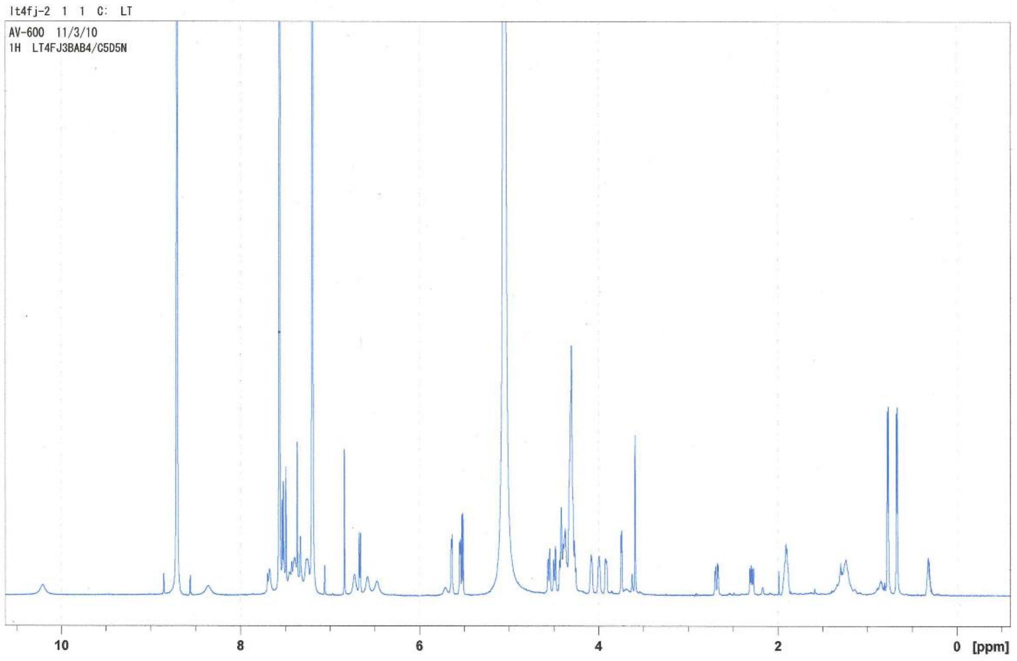

Figure S11. ^{1}H-NMR spectrum of **3**

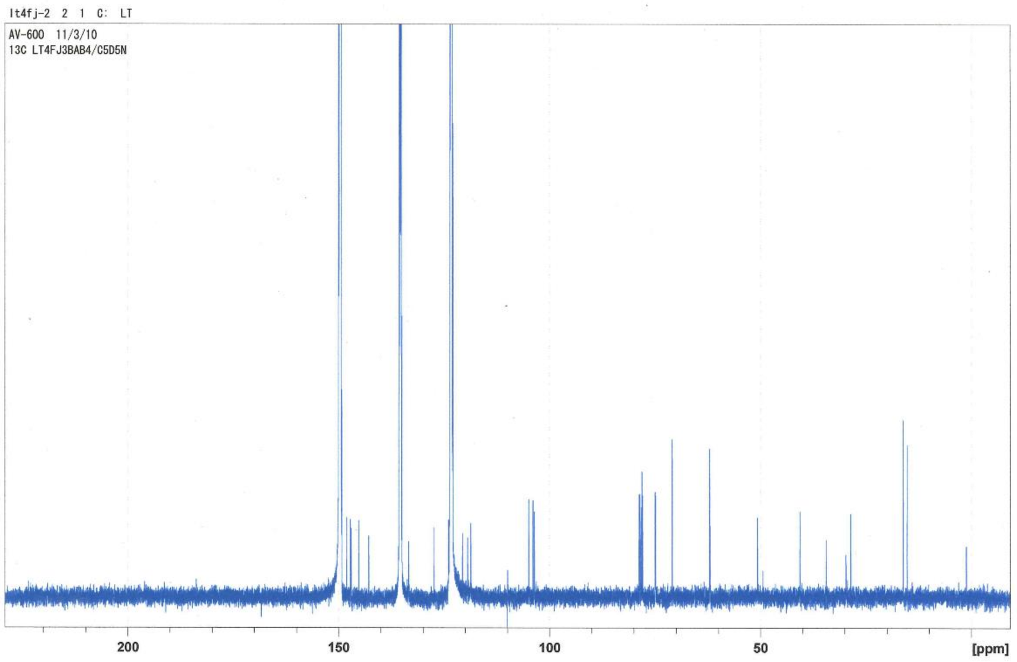

Figure S12. ^{13}C-NMR spectrum of **3**

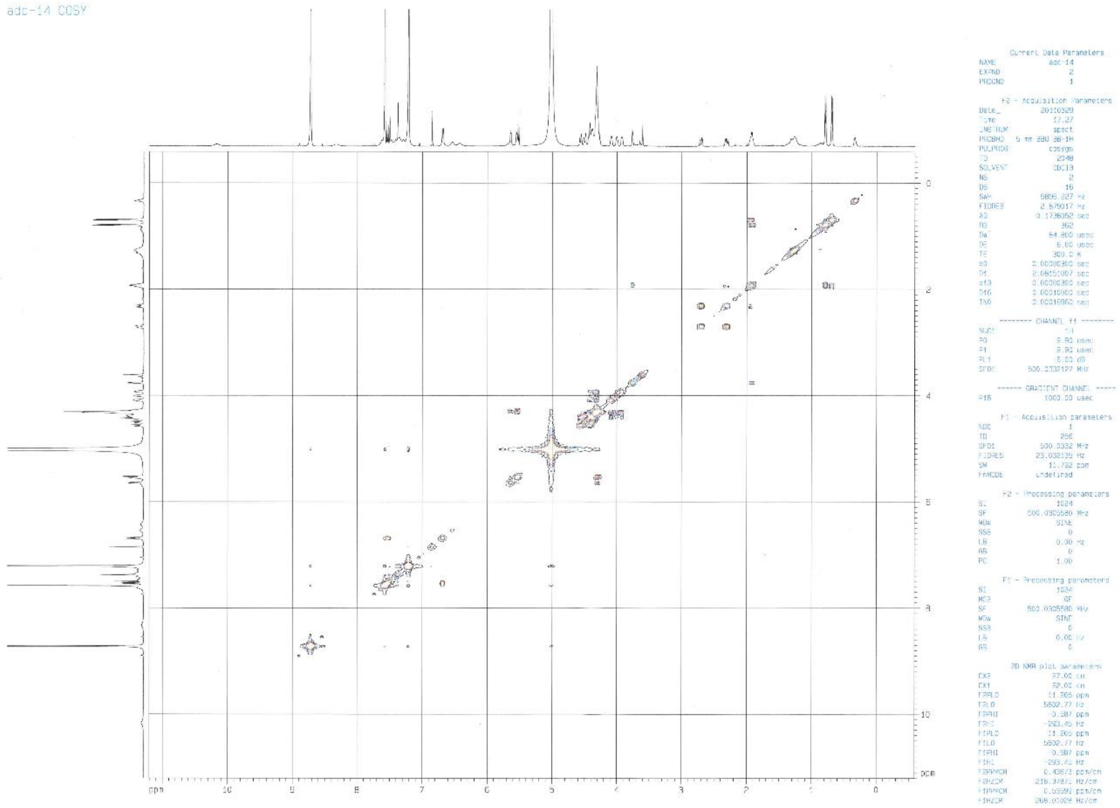

Figure S13. ^{1}H-^{1}H COSY of **3**

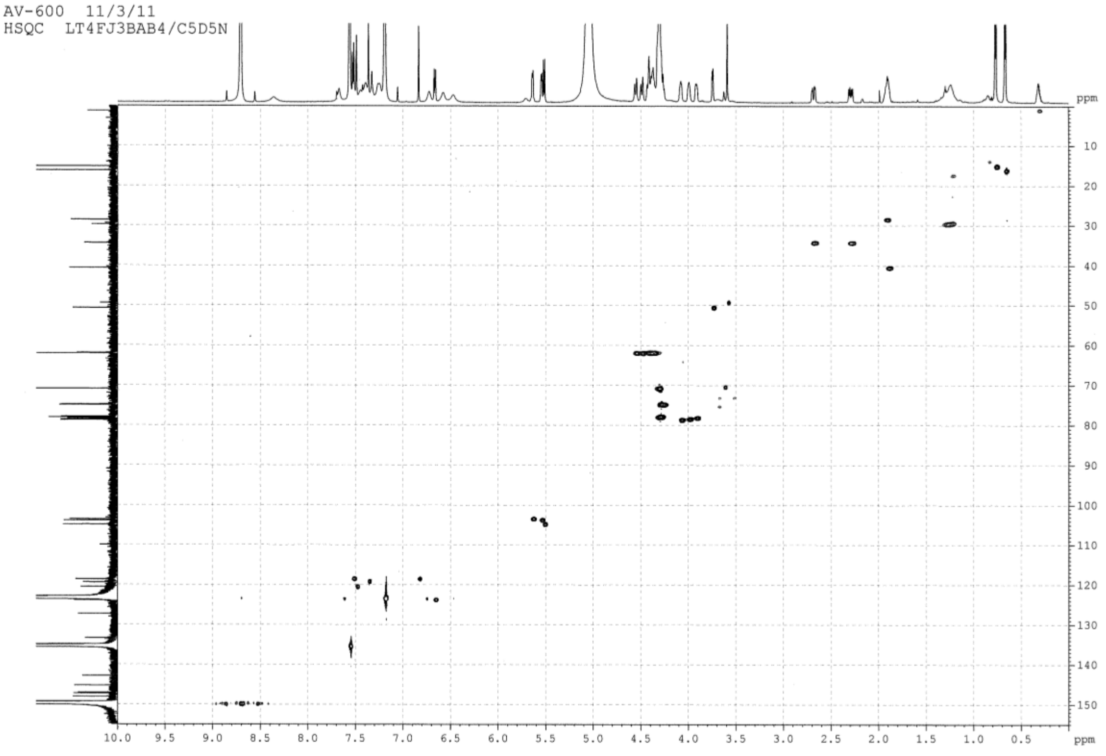

Figure S14. HSQC spectrum of **3**

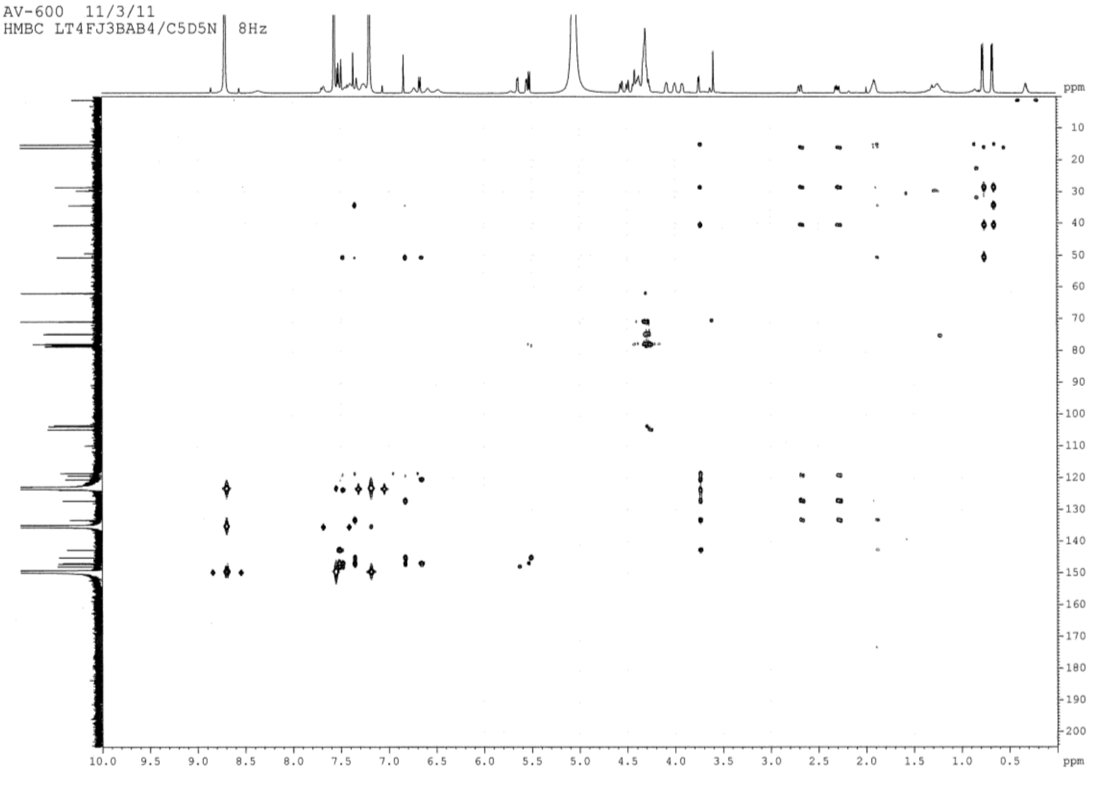

Figure S15. HMBC spectrum of **3**

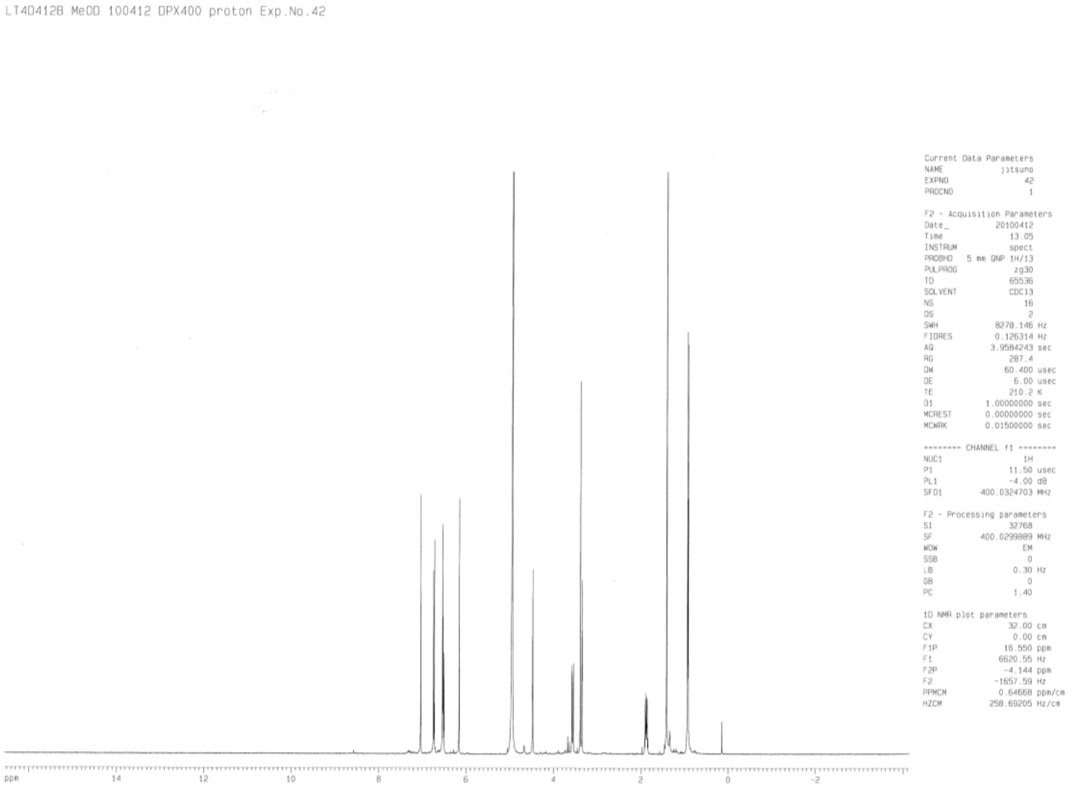

Figure S16. ^{1}H-NMR spectrum of **4**

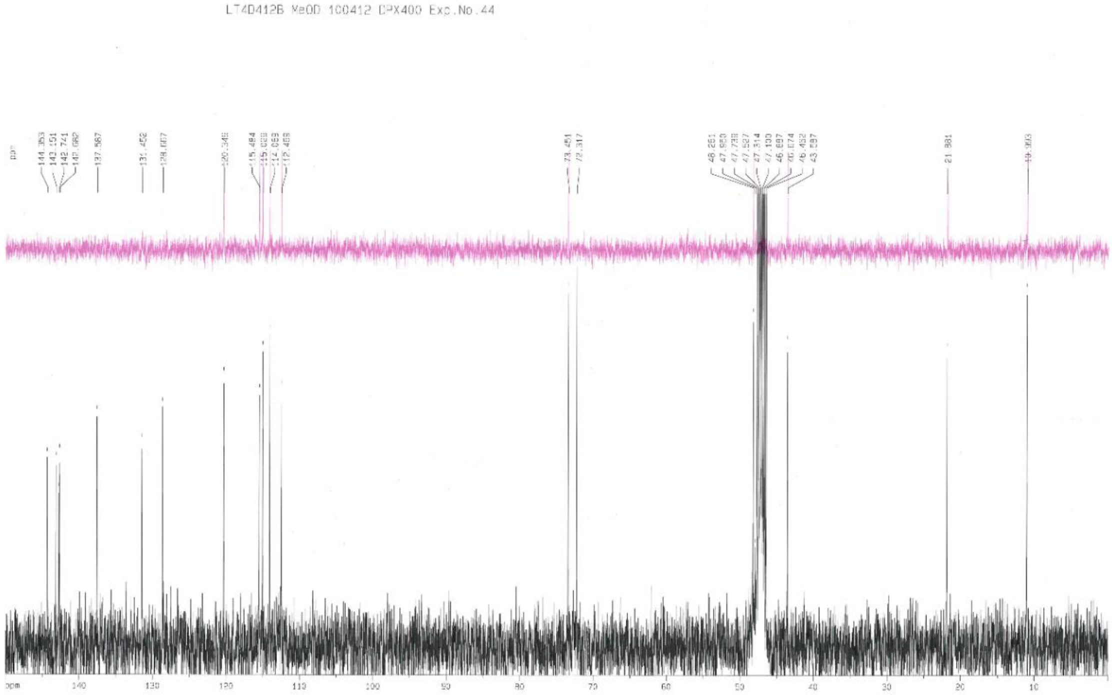

Figure S17. ^{13}C-NMR spectrum of **4**

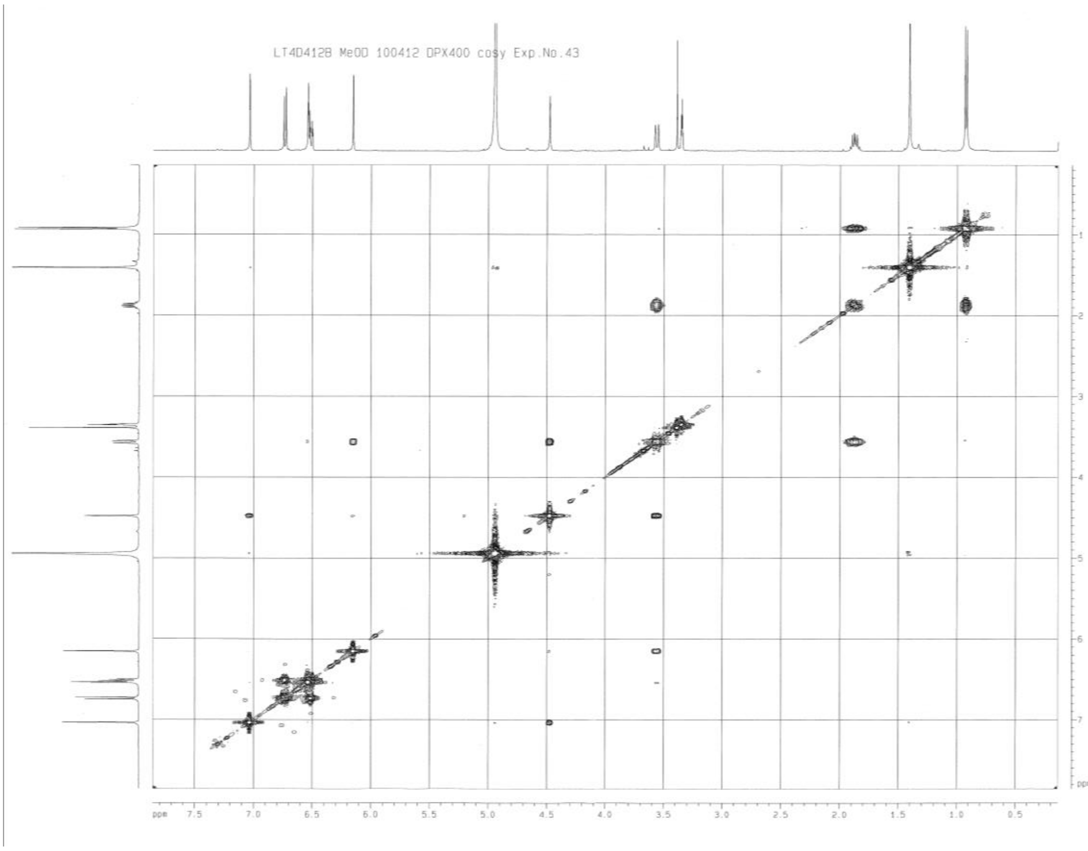

Figure S18. ^1H-^1H COSY of **4**

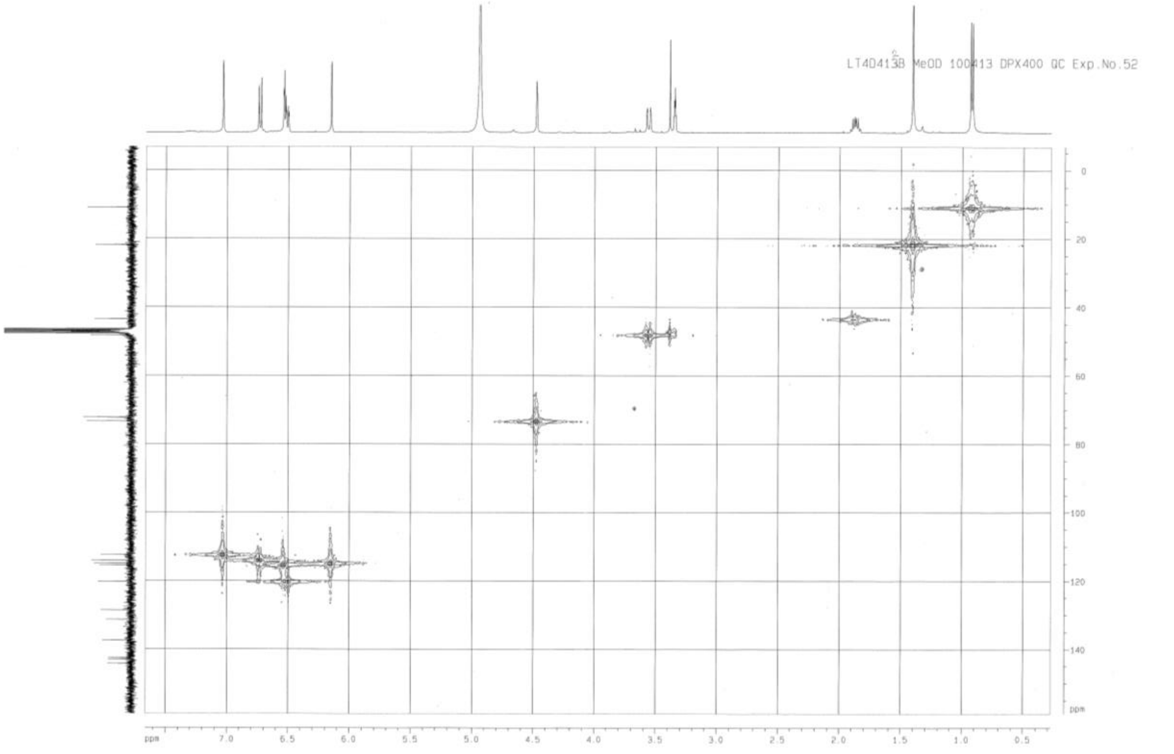

Figure S19. HSQC spectrum of **4**

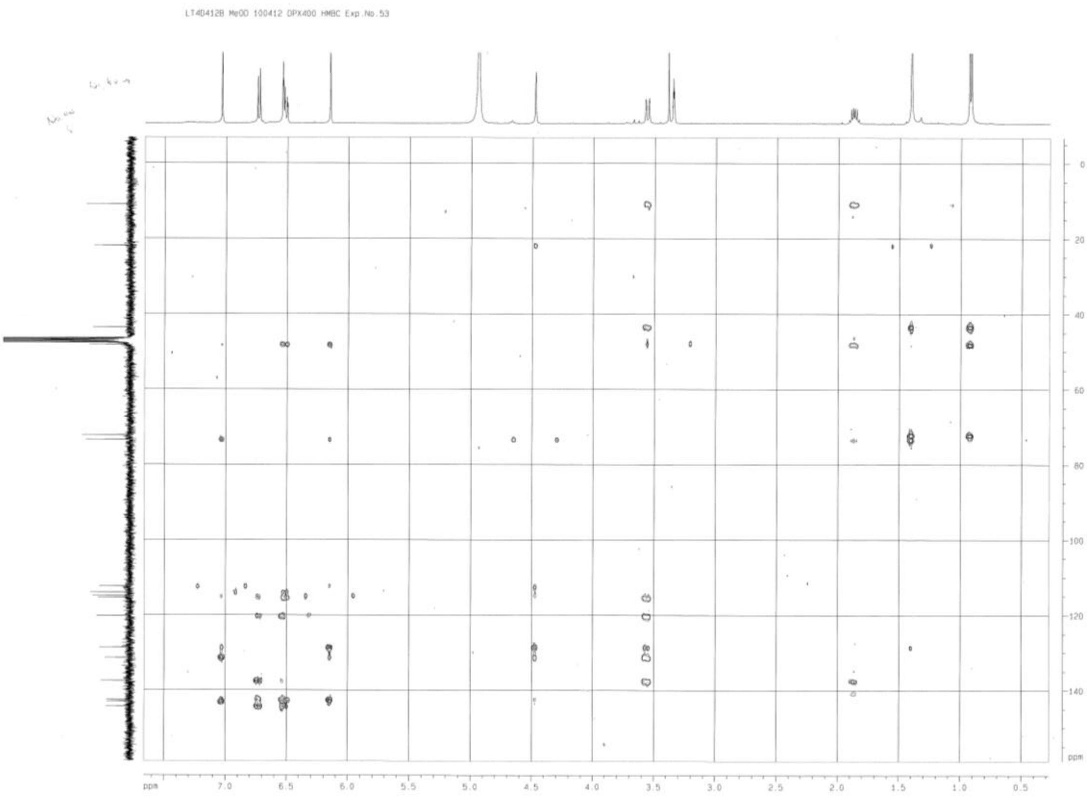

Figure S20. HMBC spectrum of **4**

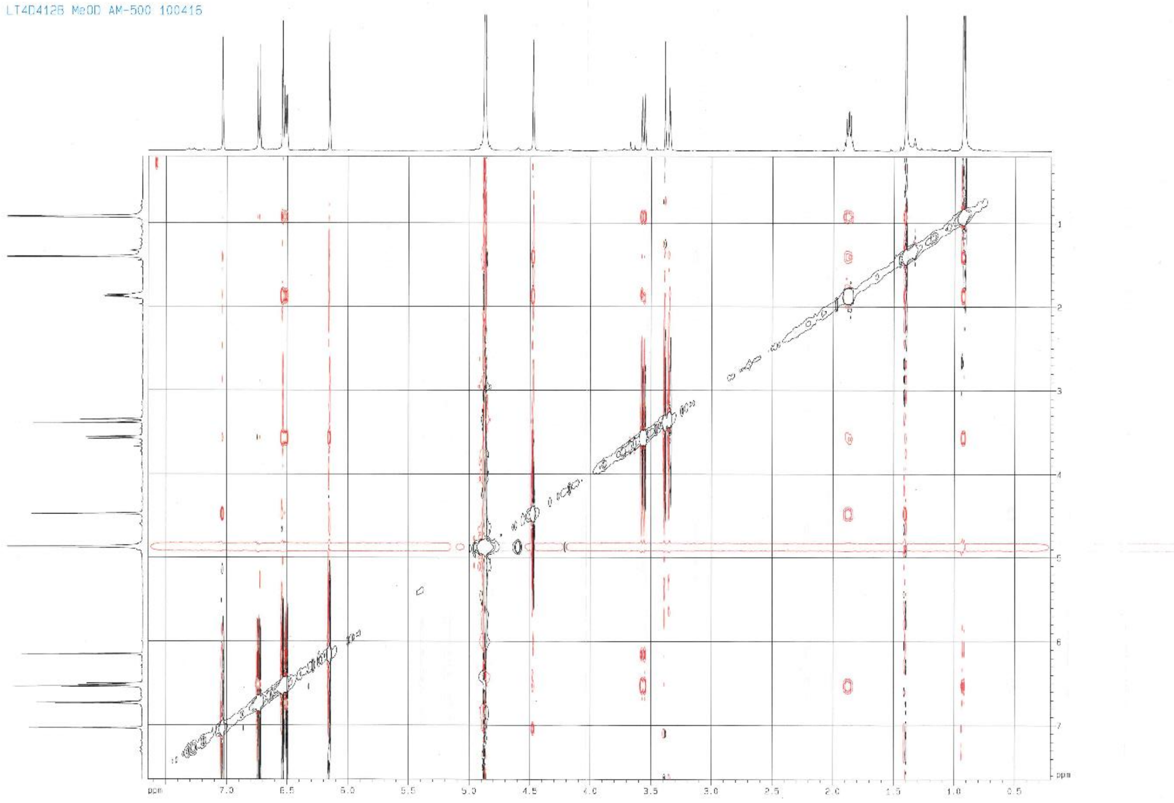

Figure S21. NOESY spectrum of **4**

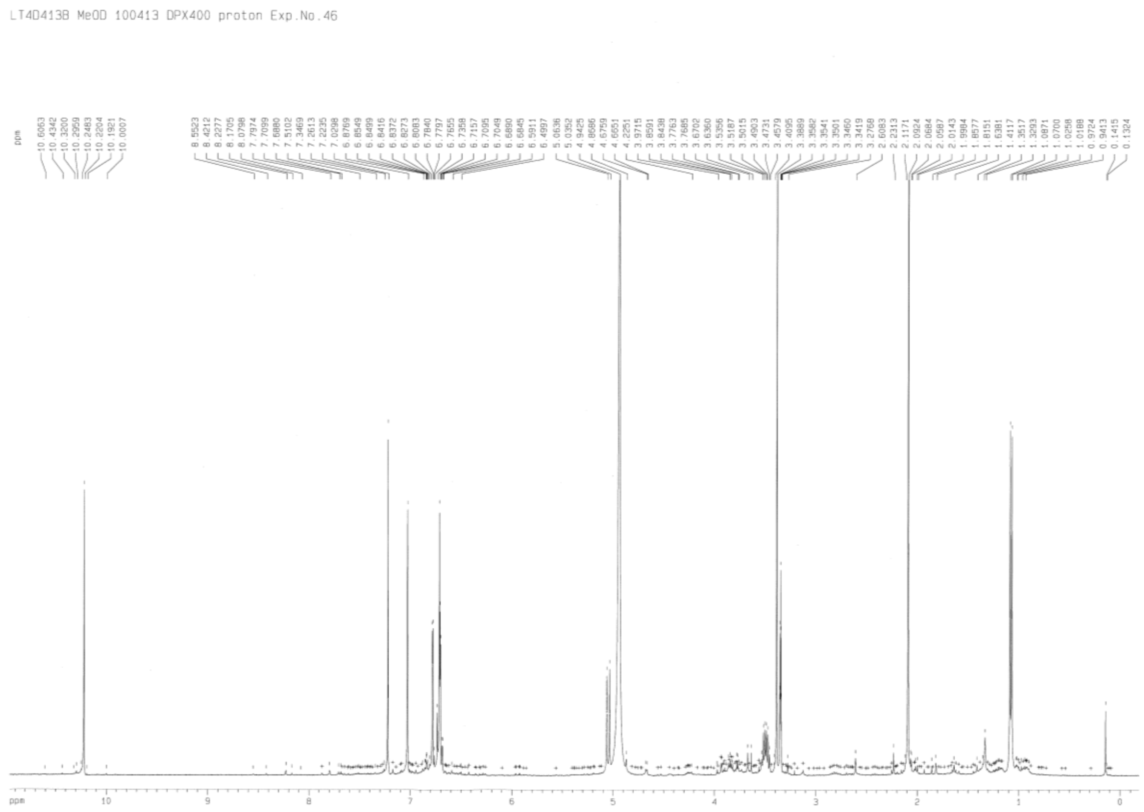

Figure S22. ^{1}H-NMR spectrum of **5**

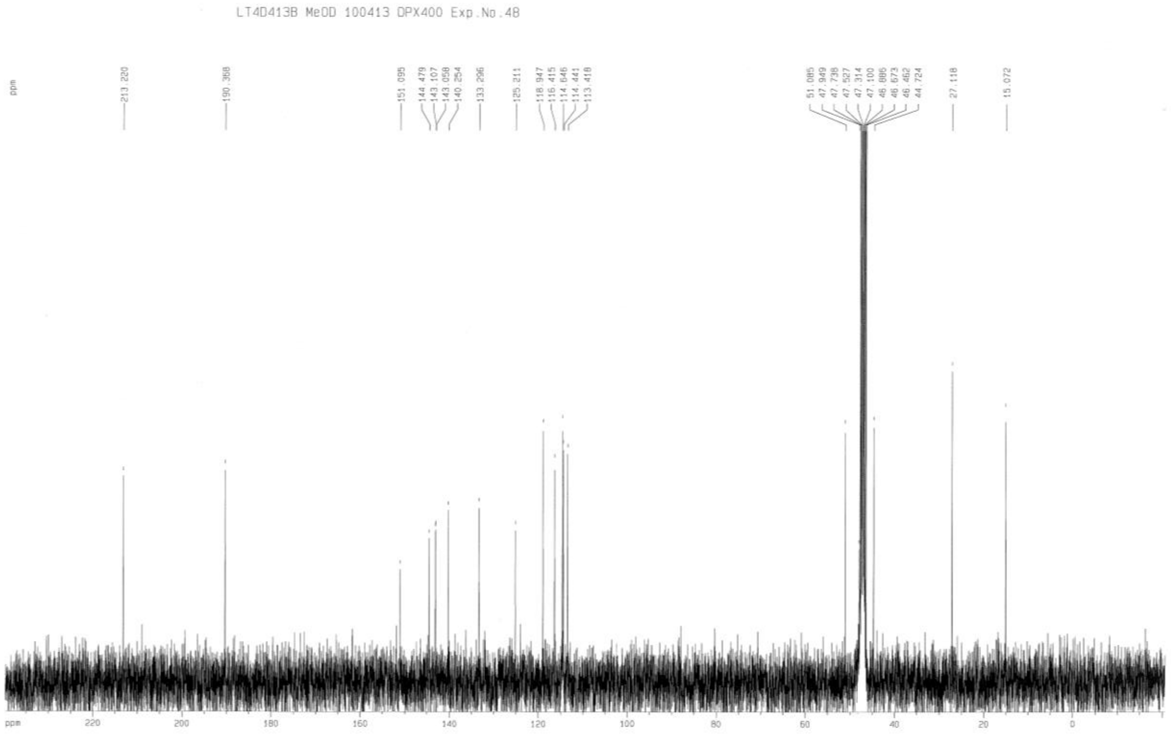

Figure S23. ^{13}C-NMR spectrum of **5**

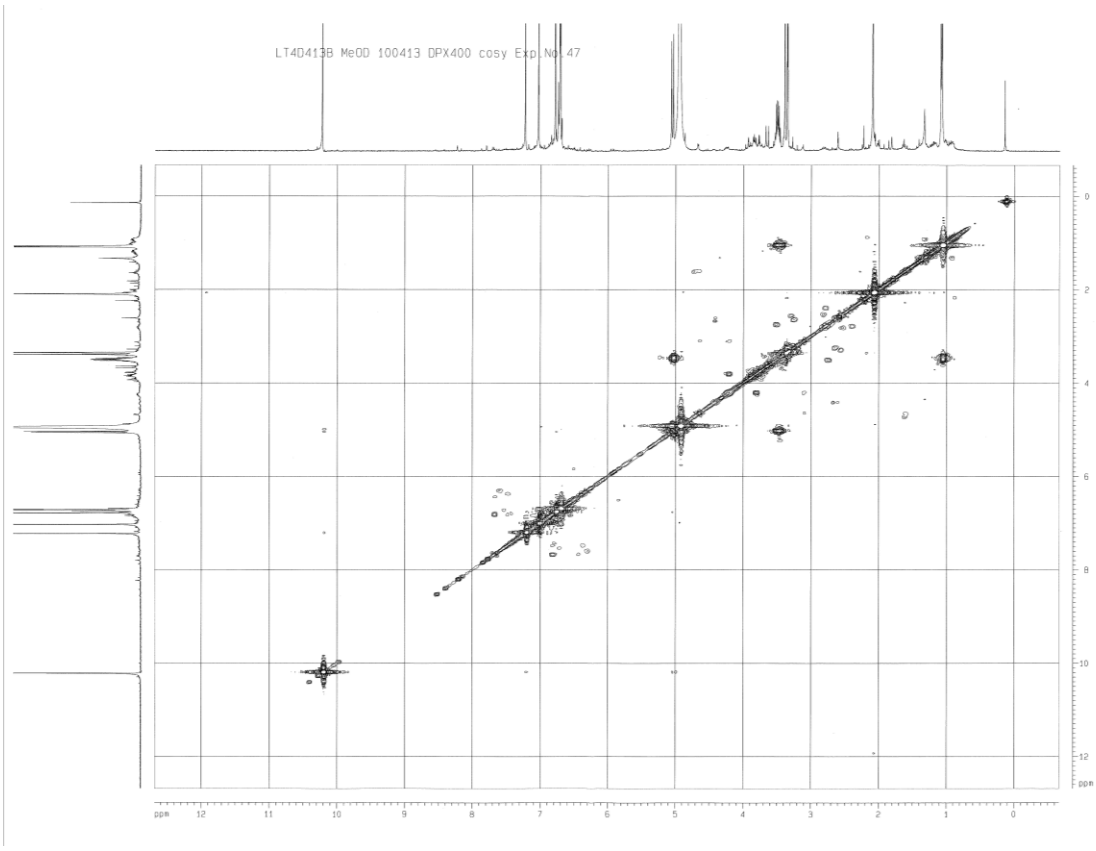

Figure S24. ^1H-^1H COSY of **5**

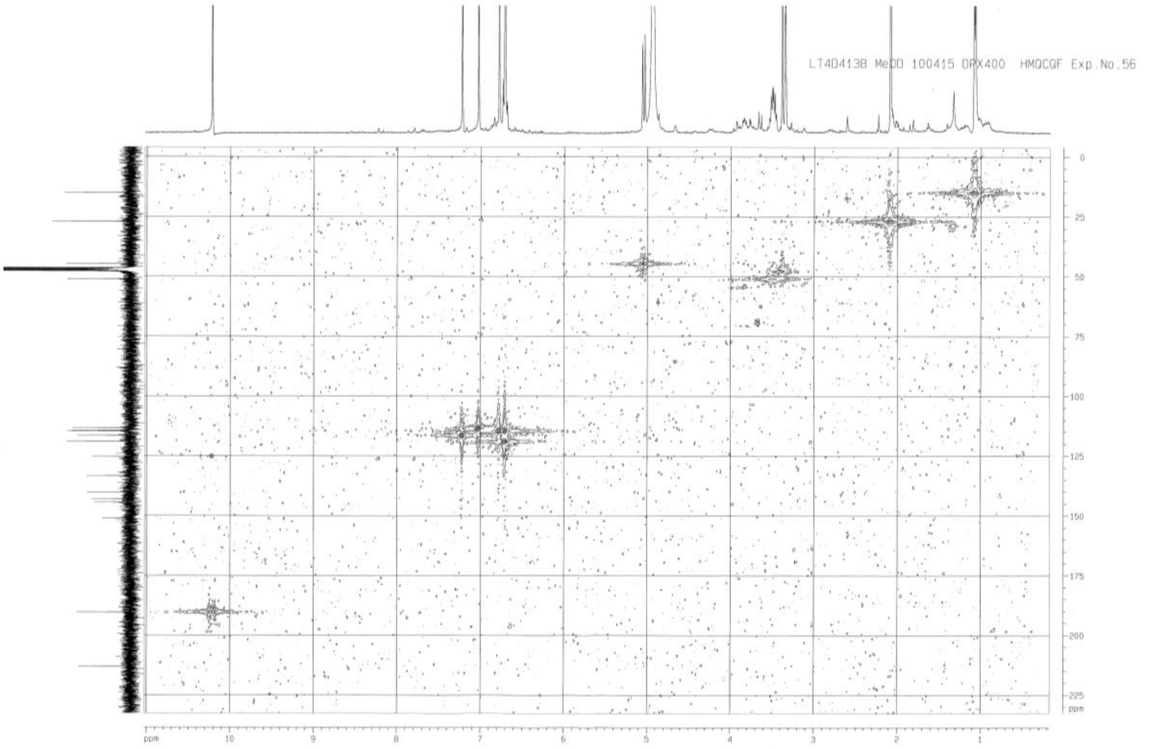

Figure S25. HSQC spectrum of **5**

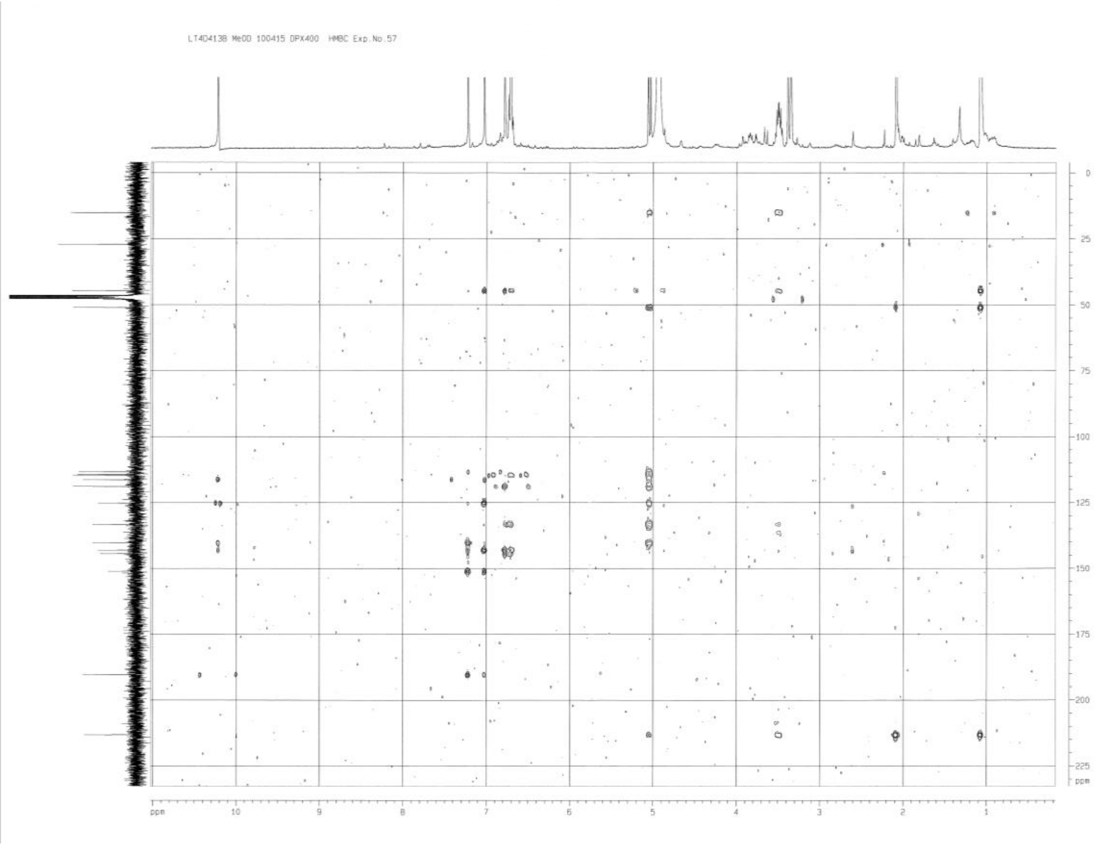

Figure S26. HMBC spectrum of **5**

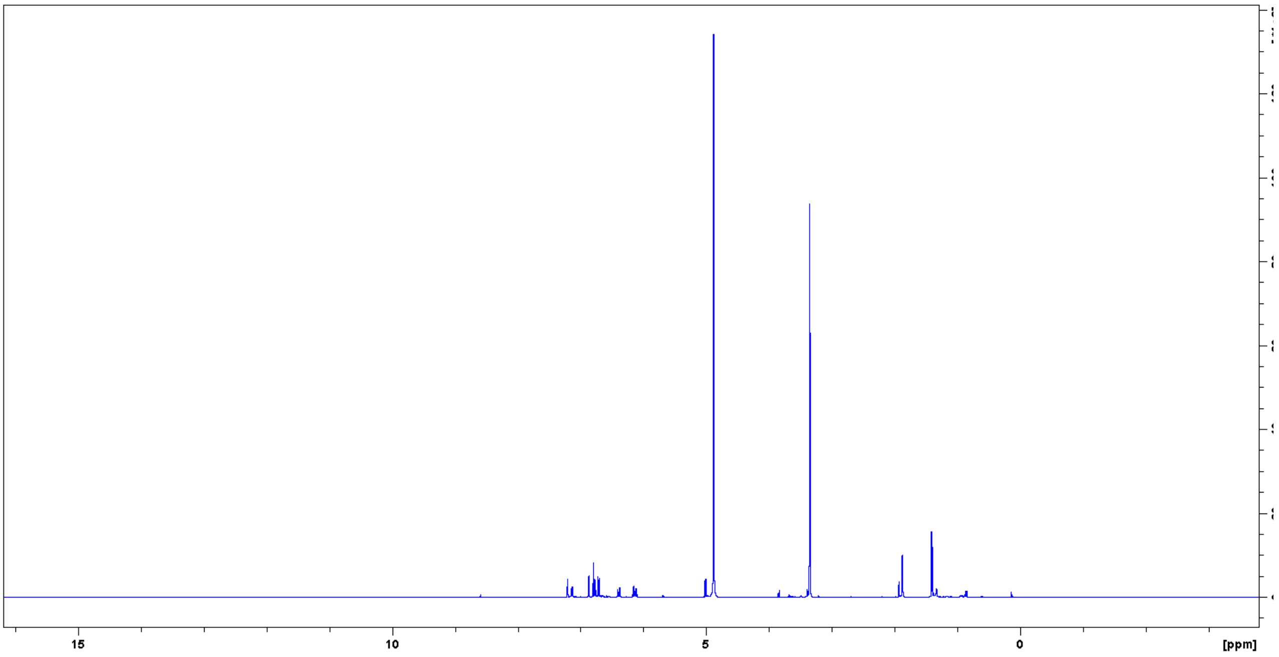

Figure S27. ^{1}H-NMR spectrum of **6**

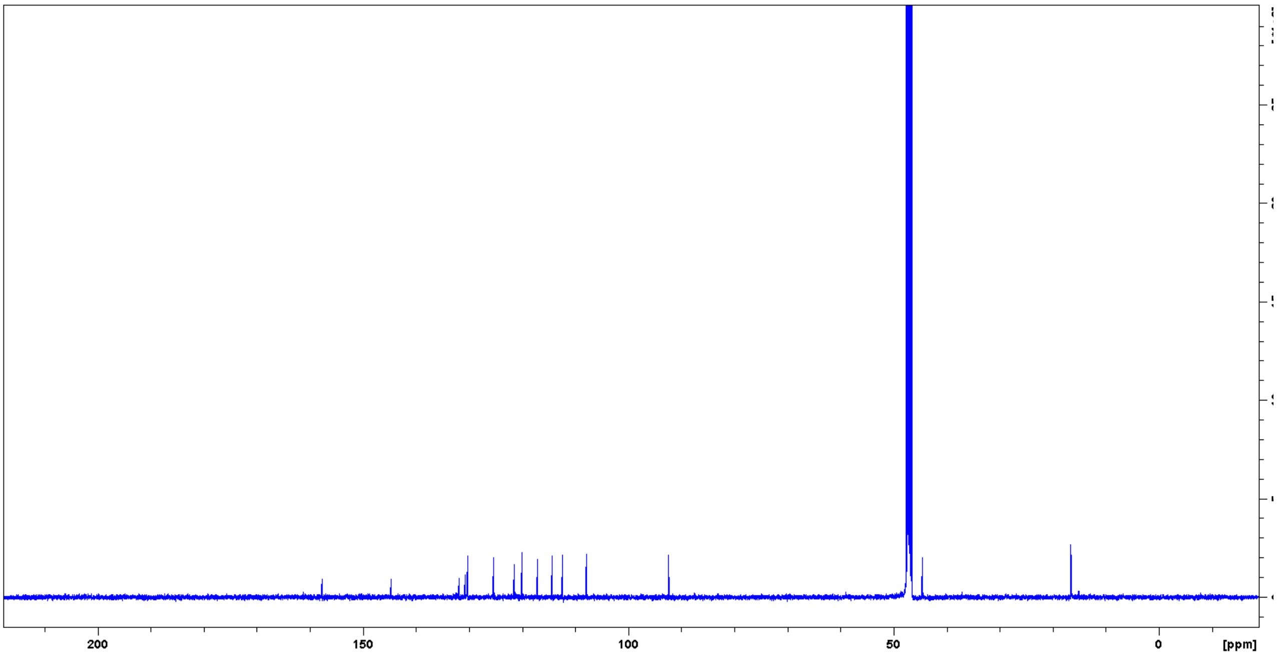

Figure S28. ^{13}C-NMR spectrum of **6**

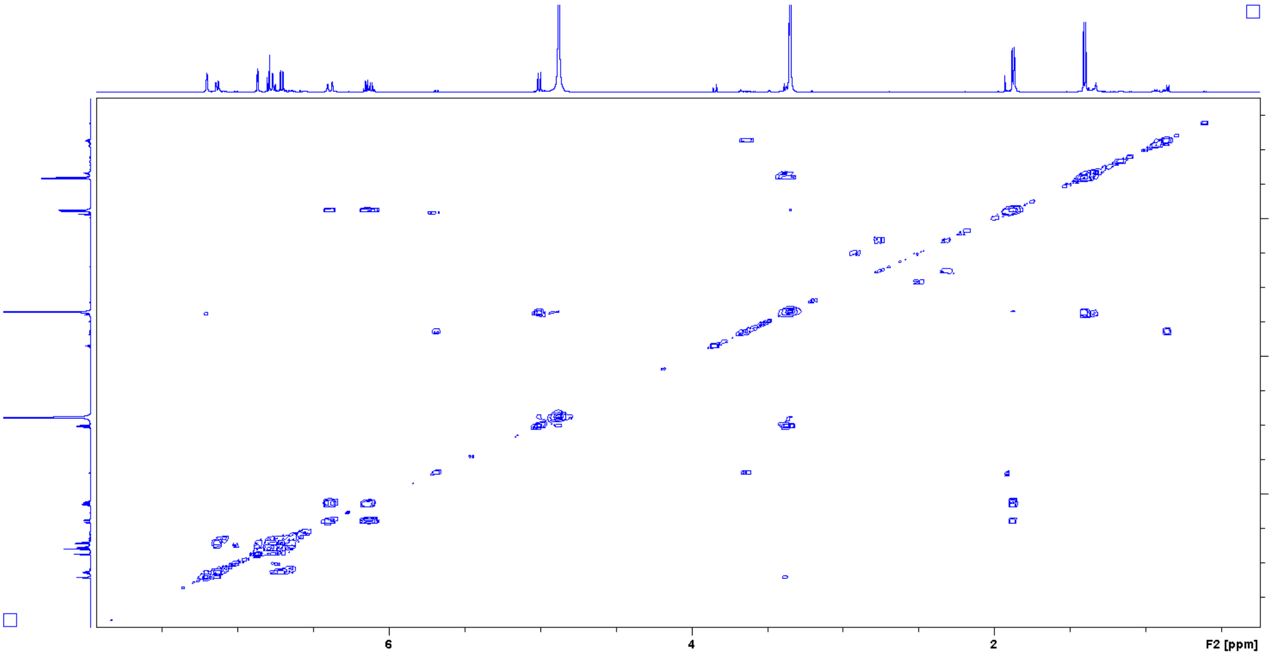

Figure S29. ^{1}H-^{1}H COSY of **6**

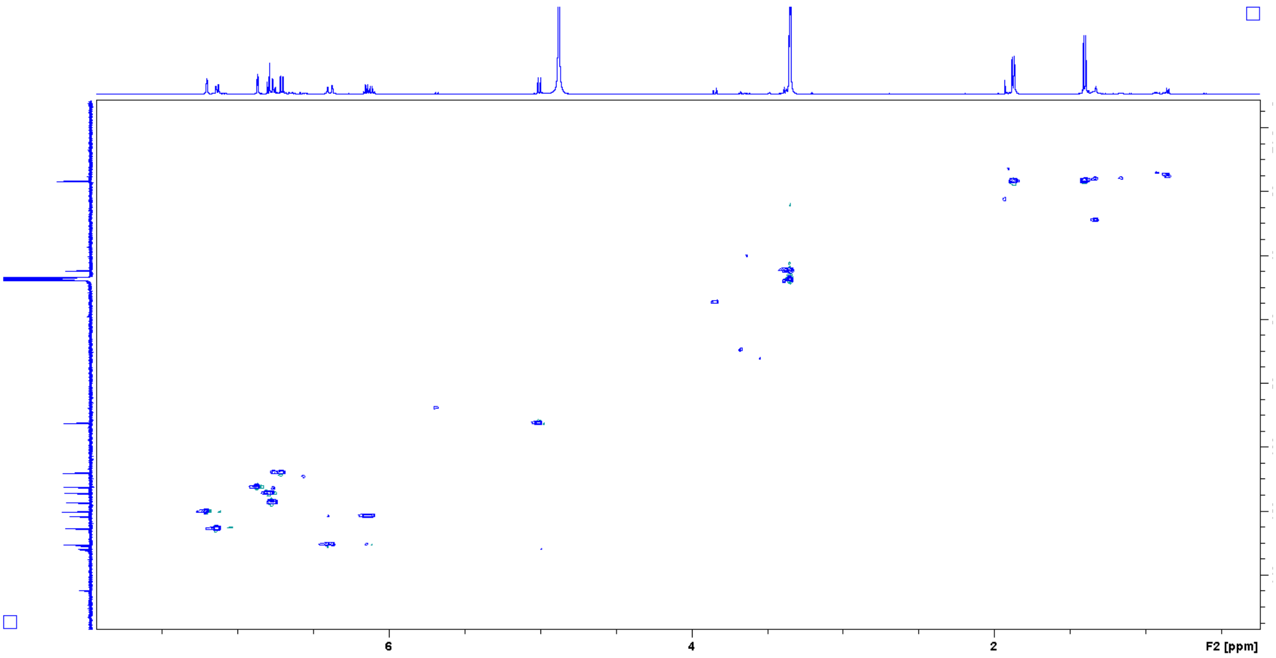

Figure S30 HSQC spectrum of **6**

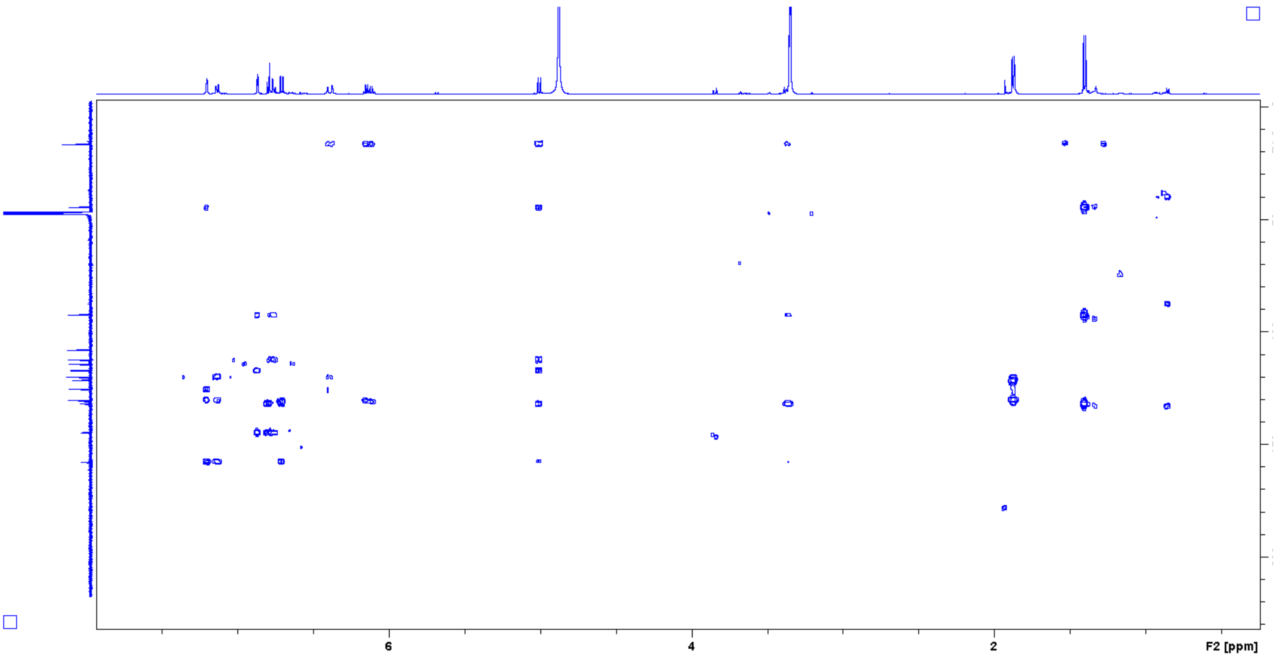

Figure S31. HMBC spectrum of **6**

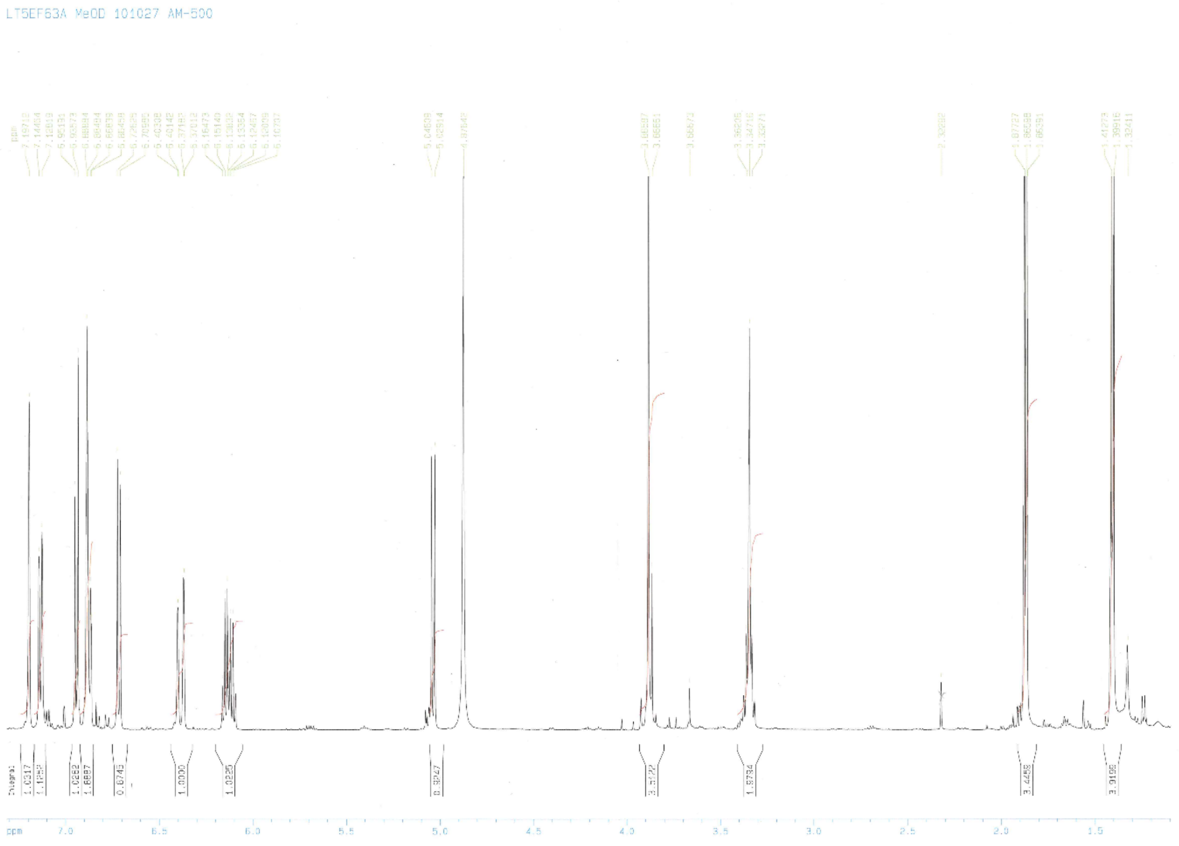

Figure S32. ^{1}H-NMR spectrum of **7**

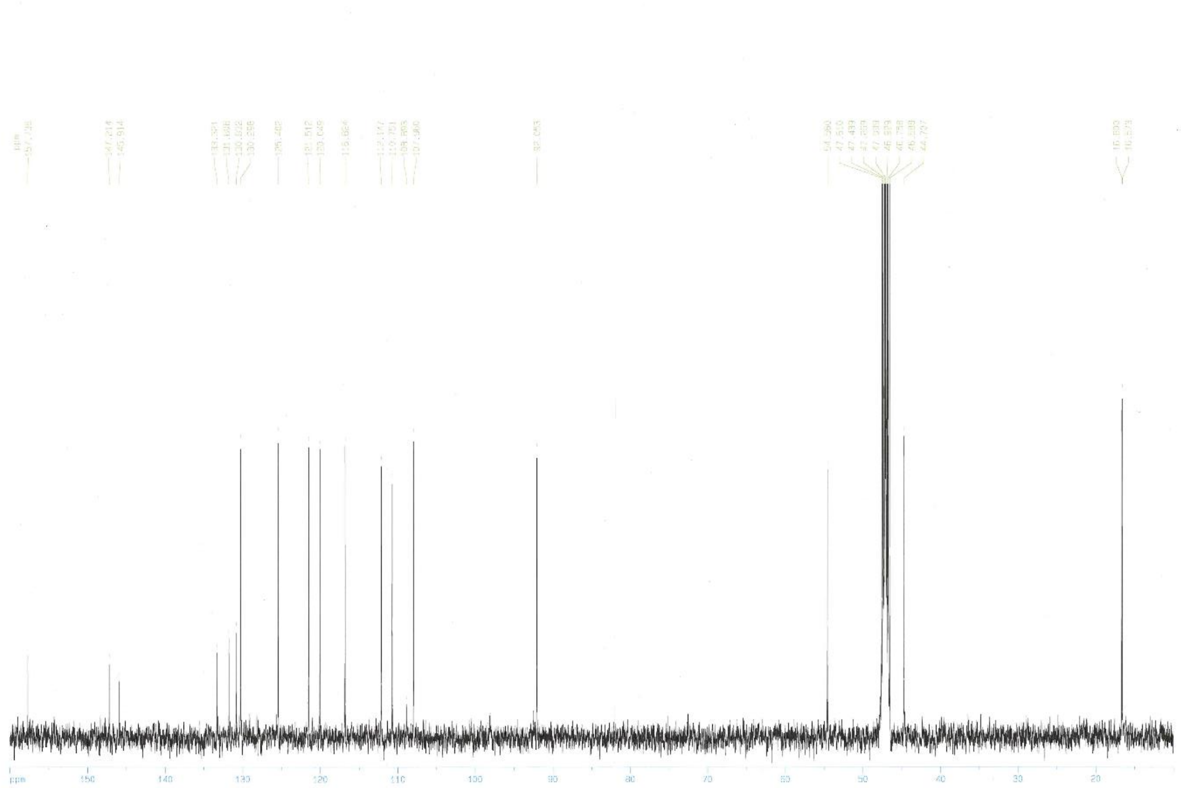

Figure S33. ^{13}C-NMR spectrum of 7

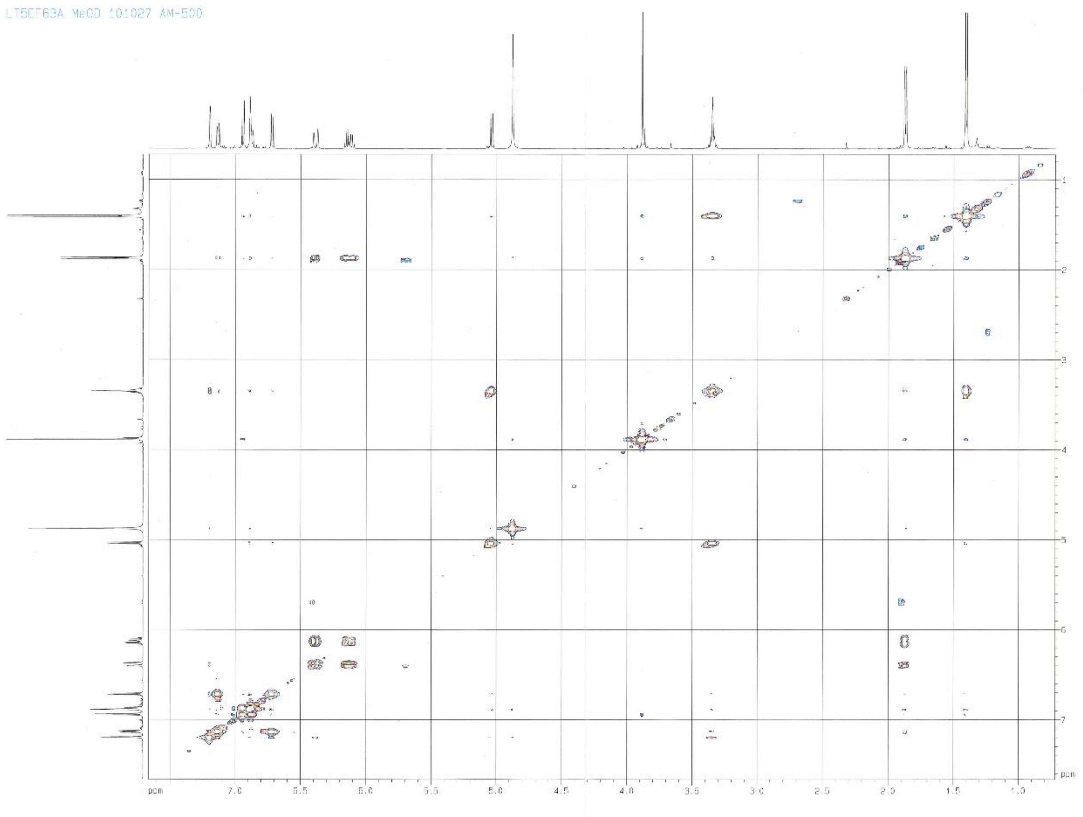

Figure S34. ^{1}H-^{1}H COSY of **7**

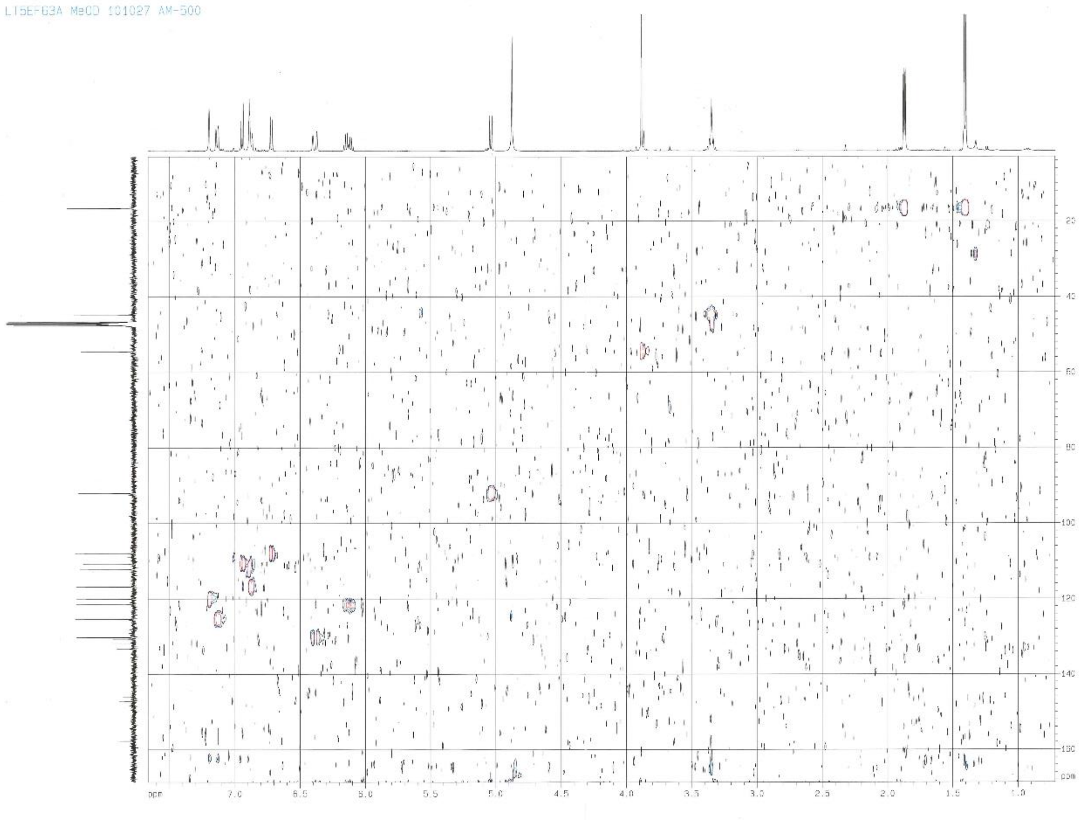

Figure S35. HSQC spectrum of **7**

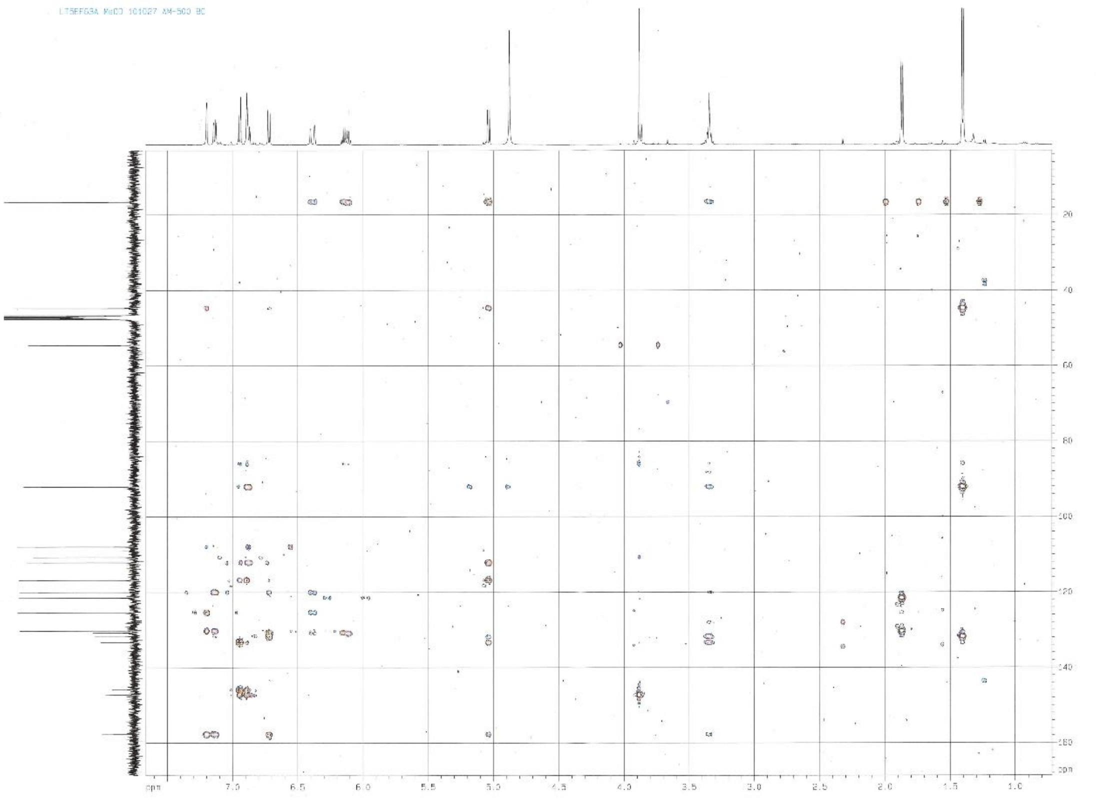

Figure S36. HMBC spectrum of **7**

J. Chem. Research (S), 1981, 27
J. Chem. Research (M), 1981, 0401–0449

Chiroptical Studies. Part 100. Lignans

PETER B. HULBERT, (the late) WILLIAM KLYNE, and P. MOLLY SCOPES*

Department of Chemistry, Westfield College, Hampstead, London, NW3 7ST

We have recorded the circular dichroism spectra of about 160 lignans of the aryl-tetralin type. These compounds show three ultraviolet absorption bands at about 280, 230, and 210 nm; the corresponding circular dichroism curves show couplets (each of one positive and one negative band) corresponding to the 280 and 210 nm absorptions and some intermediate maxima corresponding to the 230 nm u.v. absorption. The c.d. patterns are characteristic of the configurations at C-1, C-2, and C-3 in the lignan skeleton [see formula (1)] and also of the conformation of ring *B*.

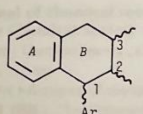

(1)

The lignans can be classified according to the relative configuration of the aryl substituent at C-1 and the alkyl substituents at C-2 and C-3 into four types (A–D), each of which occurs in the 'normal' and 'enantiomeric' forms giving a total of eight possible variations. Six of these eight types were available to us and the relationship between the configuration of these six types and the observed signs of the c.d. couplets is summarised in Table A.

Table A Signs of c.d. couplets for aryl-tetralin lignans

Type	Configuration	First couplet λ/nm	First couplet Sign	Second couplet λ/nm	Second couplet Sign
A	1β,2α,3β (*trans/trans*)	*ca.* 288–273	−	*ca.* 210–200	−
ent-A	1α,2β,3α (*trans/trans*)	*ca.* 290–275	+	*ca.* 220–210	+
ent-B	1α,2α,3β (*cis/trans*)	*ca.* 287–274	+	*ca.* 216–203	+
C	1β,2α,3α (*trans/cis*)	*ca.* 290–273	−	*ca.* 207–200	−
ent-C	1α,2β,3β (*trans/cis*)	*ca.* 288–270	+	*ca.* 209–200	+
ent-D	1α,2α,3α (*cis/cis*)	*ca.* 290–270	+	*ca.* 213–200	+

Our detailed results lead to the following conclusions, which we believe are generally valid for common types of aryl-tetralin lignans, provided that the compounds do not contain unusual substituents and that there are no conformational abnormalities.

(i) The signs of the first and second couplets near 280 and 210 nm, respectively, are the same.

(ii) The wavelengths of the maxima of the first couplet are very similar for all types, occurring at *ca.* 289 and 273 nm. The wavelengths of the second couplet are more variable, usually occurring between *ca.* 210 and 200 nm, but red-shifted by about 10 nm for some compounds, particularly those with a methylenedioxy group in ring *A*.

(iii) The sign of the first couplet reflects the configuration of the aryl substituent at C-1; 1β, negative and 1α, positive.

(iv) The relative magnitude of the two branches of the first couplet reflects the conformation of the aryl substituent at C-1 with respect to ring *B*; the longer-wavelength branch greater or smaller than the other corresponding to quasiequatorial or quasiaxial, respectively.

(v) The nature of the substituents at C-2 and C-3 in ring *B* and the presence or absence of an additional ring *B′* spanning positions 2 and 3 does not significantly affect the spectra.

(vi) Unusual substituents or an unusual substitution pattern, particularly in ring *A*, may alter the c.d. maxima significantly in sign, magnitude, or wavelength.

Techniques used: U.v., c.d.

References: 27

Table 1: Summary of *X*-ray evidence

Tables 2–5: Details of c.d. measurements

Table 6: Summary of c.d. results

*To receive any correspondence.

Paper: E/194/80 Received: 14th October 1980

Figure S37. Reference 20
